# Donor Funding for Newborn Survival: An Analysis of Donor-Reported Data, 2002–2010

**DOI:** 10.1371/journal.pmed.1001332

**Published:** 2012-10-30

**Authors:** Catherine Pitt, Joy E. Lawn, Meghna Ranganathan, Anne Mills, Kara Hanson

**Affiliations:** 1 Department of Global Health & Development, London School of Hygiene & Tropical Medicine, London, United Kingdom; 2 Saving Newborn Lives/Save the Children, Cape Town, South Africa; University of Cambridge, United Kingdom

## Abstract

**Background:**

Neonatal mortality accounts for 43% of global under-five deaths and is decreasing more slowly than maternal or child mortality. Donor funding has increased for maternal, newborn, and child health (MNCH), but no analysis to date has disaggregated aid for newborns. We evaluated if and how aid flows for newborn care can be tracked, examined changes in the last decade, and considered methodological implications for tracking funding for specific population groups or diseases.

**Methods and Findings:**

We critically reviewed and categorised previous analyses of aid to specific populations, diseases, or types of activities. We then developed and refined key terms related to newborn survival in seven languages and searched titles and descriptions of donor disbursement records in the Organisation for Economic Co-operation and Development's Creditor Reporting System database, 2002–2010. We compared results with the Countdown to 2015 database of aid for MNCH (2003–2008) and the search strategy used by the Institute for Health Metrics and Evaluation. Prior to 2005, key terms related to newborns were rare in disbursement records but their frequency increased markedly thereafter. Only two mentions were found of “stillbirth” and only nine references were found to “fetus” in any spelling variant or language. The total value of non-research disbursements mentioning any newborn search terms rose from US$38.4 million in 2002 to US$717.1 million in 2010 (constant 2010 US$). The value of non-research projects exclusively benefitting newborns fluctuated somewhat but remained low, at US$5.7 million in 2010. The United States and the United Nations Children's Fund (UNICEF) provided the largest value of non-research funding mentioning and exclusively benefitting newborns, respectively.

**Conclusions:**

Donor attention to newborn survival has increased since 2002, but it appears unlikely that donor aid is commensurate with the 3.0 million newborn deaths and 2.7 million stillbirths each year. We recommend that those tracking funding for other specific population groups, diseases, or activities consider a key term search approach in the Creditor Reporting System along with a detailed review of their data, but that they develop their search terms and interpretations carefully, taking into account the limitations described.

*Please see later in the article for the Editors' Summary*

## Abstract 2

With recent increases in development assistance money for maternal and child health, Catherine Pitt and colleagues examine whether foreign aid specifically for newborns has changed, whether it's on par with the burden of newborn deaths worldwide, and how such funding can be tracked.

## Editors' Summary

**Background:**

In 1990, 12 million children—most of them living in developing countries—died before they reached their fifth birthday. Faced with this largely avoidable loss of young lives, in 2000, world leaders set a target of reducing under-five mortality (deaths) to one-third of its 1990 level by 2015 as Millennium Development Goal 4 (MDG4); this goal, together with seven others, aims to eradicate extreme poverty globally. In recent years, progress towards reducing child mortality has accelerated but remains insufficient to achieve MDG4, in part, because progress towards reducing neonatal mortality—deaths during the first 28 days of life—has been particularly slow. Neonatal deaths now account for a greater proportion of global child deaths than in 1990—43% of the 7 million children who died before their fifth birthday in 2011 died during the neonatal period. The major causes of neonatal deaths are complications of preterm and term delivery and infections. Simple interventions such as improved hygiene at birth and advice on breastfeeding can substantially reduce neonatal deaths.

**Why Was This Study Done?:**

To achieve MDG4, more must be done to prevent deaths among newborn babies. One reason that progress in reducing neonatal mortality is slow could be insufficient donor funding (aid) for newborn health. Previous analyses by, for example, Countdown to 2015 (which tracks coverage levels for health interventions that reduce maternal, newborn, and child mortality) indicate that donor funding has increased for maternal, newborn, and child health over the past decade, but how much of this aid directly benefits newborns is unknown. Here, the researchers develop a method for tracking aid flows for newborns and examine changes in this flow over the past decade by applying their new strategy to the Organisation for Economic Co-operation and Development (OECD) Creditor Reporting System (CRS) Aid Activity database. This database collects information about official development assistance for health given (disbursed) to developing countries by member countries of the OECD Development Assistance Committee, international organizations, and some private donors.

**What Did the Researchers Do and Find?:**

The researchers developed a comprehensive set of search terms related to newborn survival by piloting it on the Countdown to 2015 official development assistance database, which covers the years 2003–2008. They then used their list of 24 key terms to search the CRS database from 2002 (the first year for which relatively complete disbursement data are available) to 2010 (the most recent year for which data are available) and classified each retrieved project according to whether its funding activities aimed to benefit newborns exclusively or to improve the health of other population groups as well. The researchers found that key terms related to newborns were rare in disbursement records before 2005 but that their frequency increased markedly thereafter. The total value of non-research disbursements (aid provided for programmatic or advocacy activities) that mentioned any newborn search terms increased from US$38.4 million in 2002 to US$717.1 million in 2010. The value of non-research projects that exclusively benefitted newborns fluctuated; in 2010, it was $US5.7 million. Finally, the US and United Nations Children's Fund (UNICEF) provided the largest value of non-research funding mentioning newborns and exclusively benefitting newborns, respectively.

**What Do These Findings Mean?:**

These findings indicate that the value of aid disbursements mentioning newborns or an activity likely to benefit newborns increased 20-fold between 2002 and 2010 and constituted an increasing proportion of aid for maternal, newborn, and child health. Although this increase may partly reflect increased detail in aid disbursement reporting, it is also likely to reflect an increase in donor attention to newborn survival. The accuracy of these findings is likely to be affected by limitations in the search strategy and in the CRS database, which does not capture aid flows from emerging donors such as China or from many private foundations. Moreover, because these findings take no account of domestic expenditure, they do not provide a comprehensive estimate of the value of resources available in developing countries for newborn health. Nevertheless, investment in newborn survival is unlikely to be commensurate with global newborn mortality. Thus, an expansion of programmatic funding from donors as well as increased governmental support for newborn health in developing countries is urgently needed to catalyze the scale-up of cost-effective interventions to save newborn lives and to meet MDG4.

**Additional Information:**

Please access these Web sites via the online version of this summary at http://dx.doi.org/10.1371/journal.pmed.1001332.

- The United Nations Childrens Fund (UNICEF) works for children's rights, survival, development, and protection around the world; it provides information on Millennium Development Goal 4 and its Childinfo website provides detailed statistics about child survival and health, including the 2012 report of UN Inter-agency Group of Child Mortality Estimation; its Committing to Child Survival: a Promise Renewed webpage includes links to its 2012 progress report, to a video about progress made in reducing child deaths worldwide, and to stories about child survival in the field
- The World Health Organization has information about Millennium Development Goal 4 and about maternal, newborn, child, and adolescent health (some information in several languages)
- Countdown to 2015 provides additional information on maternal, newborn, and child survival, including its 2012 report Building a Future for Women and Children
- The Healthy Newborn Network (HNN) is a community of more than 70 partner organizations addressing critical knowledge gaps for newborn health providing recent data on newborn survival and analyses of country programs
- Information on and access to the Organisation for Economic Co-operation Development Creditor Reporting System Aid Activities database is available
- Further information about the Millennium Development Goals is available

## Introduction

Efforts to reduce maternal mortality and reach the Millennium Development Goal (MDG) 5A have received increased attention in recent years and the maternal mortality ratio is now falling at around 3.1% per year [1],[2]. The rate of reduction in post-neonatal child mortality (children aged 1–59 mo) has also accelerated over the last decade and is now declining at over 2.9% per year [3]. In contrast, the neonatal mortality rate (children aged 0–28 d) is declining, but at only 2.1% [4], and so newborns account for an increasing proportion of under-five deaths [5]. Estimates for 2011 indicate that newborns account for 43% of child deaths worldwide and 52% of child deaths in South Asia [5]. Addressing neonatal mortality is therefore key to achieving MDG 4 for child survival.

A range of well-known interventions are effective in improving newborn survival, feasible even in challenging health system contexts [6] and likely to be highly cost-effective [7], yet largely remain at low coverage [8]. While a focus on the continuum of care is important [9], the lagging progress in neonatal mortality at a global level and the failure even to set a goal to reduce stillbirths [10] suggest that newborns are in need of particular attention [8]. In 2010, Shiffman provided qualitative evidence of an increase in attention for newborn survival on the global health agenda over the previous 10 y, and especially since 2005 [11]. He provided ample evidence of the increasing focus on newborns in global discussions; however, there was little systematic evidence of the extent to which this attention translated into resources.

The Commission on Information and Accountability for Women's and Children's Health is the most recent in a series of efforts to promote accountability of donors and developing country governments for improving health, including that of the newborn. Recognising the importance of resource tracking, they have endorsed existing efforts to monitor maternal, newborn, and child health funding (MNCH), although none of the existing exercises distinguish funding that would benefit newborns from that benefitting mothers or older children [12],[13]. According to analyses by the Countdown to 2015 [12],[14],[15] and the Institute for Health Metrics and Evaluation (IHME) [13],[16],[17], donor funding has increased for MDG 4 and MDG 5A. Attention to newborn survival is more recent and no analysis to date has attempted to disaggregate the value of aid directly benefitting newborns, although policy makers have expressed interest in doing so [18].

Our objectives were to determine if and how aid flows for newborn health can be tracked, to examine changes in the last decade, and to consider methodological implications tracking funding for other specific population groups, diseases, or types of activities. We first provide an overview of relevant data sources, previous analytical approaches, and our methods, then present the results of our case study, and finally discuss the implications and conclusions of our findings for newborn survival policy and programmes in particular and then for other analyses of funding for a specific condition or target group.

## Data Sources

The Creditor Reporting System (CRS) aid activity database is the most accepted, consistent, and complete database available for official development assistance (ODA) for health [19]. Furthermore, the Commission on Information and Accountability exhorted all major donors, whether public or private, to report to the CRS and encouraged further exploitation of these data [20]. The CRS is maintained and administered by the Organisation for Economic Co-operation and Development (OECD). All members of the Development Assistance Committee (DAC) of the OECD report their ODA on an annual basis to the CRS, while a number of organisations, including the European Commission, most United Nations (UN) bodies, the Bretton Woods institutions, and the largest global health initiatives, report voluntarily. In the past several years, the quality and quantity of its data have improved significantly. Additional donors have also begun reporting (UNDP, South Korea, WHO, GAVI, Global Fund, the Gates Foundation, the United Arab Emirates, Kuwait, and the Organization of the Petroleum Exporting Countries' Fund for International Development [OFID]) and existing donors have improved the completeness and accuracy of their reporting.

According to the CRS guidelines, which are agreed by all OECD donors, each record within the aid activity database should reflect a single aid activity, be it a project, programme, or funding to an organisation or government, in a single year [21]. Donors provide data for each record on the value and year of the disbursement or commitment, the title, a short description, and a long description of the aid activity being funded. The growth in aid and the increasing tendency of some donors to report each transaction (rather than aggregating all transactions to a particular recipient in the same year and purpose code) have resulted in a very large and rapidly growing database, which contained 247,650 ODA records for 2010 alone.

Despite recommendations that the CRS should allow records to reflect multiple purposes [22], donors must currently categorise each of their records under a single sector code and a single, more specific purpose code relating to the activity as a whole, or, if the activity targets multiple purposes, the main purpose of the funding. Within the health and population sectors, purpose codes allow projects to be categorised according to one of several health systems areas such as “basic health infrastructure” or “health personnel development” or to one of several disease-specific areas such as malaria or HIV/AIDS, but none of the codes except for “reproductive care” allow for categorisation based on the population group targeted. Furthermore, purpose codes are not mutually exclusive and many activities benefit multiple purposes and population groups. For example, it is unclear whether a project to address malaria in pregnancy, a cause of preterm delivery, low birth weight, and possibly stillbirths, should be categorised as a malaria project or a reproductive health project. Hence, purpose codes cannot accurately reflect the value of aid benefitting even those purposes for which codes currently exist.

In addition to the CRS aid activities database, several other primary and secondary sources of project-level data exist and are reviewed elsewhere [19],[23]. Other than the Financial Tracking Service, which collects data on humanitarian aid directly from donors, and individual donors' databases of their own aid, the remaining project-level databases, including both the IHME's Development Assistance for Health Database (Country and Regional Recipient Level) and the Countdown to 2015's Database of Official Development Assistance, build directly on the CRS. IHME's project-level database covers the years 1990–2008; its data for bilateral donors and the European Commission are based on analyses of the CRS database, while its data for multilateral institutions and the Gates Foundation are based on information obtained from a variety of other sources [17]. The Countdown database covers the years 2003–2008 and differs from the full CRS in that it excludes research activities, but includes data provided directly by GAVI for 2003–2007, as these data were not available in the CRS.

## Previous Analytical Approaches for Tracking Aid for Specific Target Groups or Diseases

Three main analytical approaches have been employed to disaggregate health funding within the CRS: (1) use of the existing CRS sector and purpose codes, (2) key term searches of the text fields in the database, and (3) individual recoding of each record based on a predefined framework. Each of these approaches has particular strengths and weaknesses (Table 1). These approaches can be applied to either the commitment or the disbursement value, which also have advantages and disadvantages (Table 2). In some cases, additional data sources, such as the Financial Tracking Service [24] and data provided directly by the donor or agency [12],[14]–[16], have been used to complement the CRS (Table 3).

**Table 1:** Strengths and weaknesses of the three main analytical approaches for analyzing health aid to specific areas.

Analytical Approach	Strengths	Weaknesses
Existing CRS sector and purpose codes	Time-efficient to implement	Purpose codes are subject to interpretation and misreporting by donors
	Easily replicable	Purpose codes were not designed to assess health aid according to beneficiary group
	Based on codes that donors themselves accept	Purpose codes are not conceptually mutually exclusive
	Re-analysis of updated data sources is relatively easy	Purpose codes for multi-purpose activities only reflect the largest activity
Key term searches in titles and descriptions	Time-efficient to implement as relies on an automated approach	Reporting bias based on how donors describe projects
	Easily replicable	Does not allow for misclassification of projects or more granularity in disbursement detail without careful scrutiny of project descriptions
	Re-analysis of updated CRS databases is relatively easy	May lack sensitivity and/or specificity if search terms are not developed carefully or cannot be identified
		Open to gaming/manipulation
Coding of individual records based on a predefined framework	Allows explicit and comprehensive estimate based on all the information available in the database (title, description and purpose code)	Labour-intensive
	Can address donor errors in assigning purpose codes, spelling mistakes, and use of key terms in describing the situation rather than the funded activities	Replication possible, but time consuming
		Codes are subject to interpretation and misreporting by analysts
		Results may be less accepted by donors
		Updated or revised CRS databases cannot be combined with completed analytic databases easily unless the specific changes are identified

**Table 2:** Strengths and weaknesses of the two possible measures for analyzing health aid to specific areas.

Strengths/Weaknesses	Measure		
	Commitment	Disbursement	Both
Strengths	Relatively complete data available from 1996	Reflects the actual value of funding made available to a recipient in a given year	Maximizes the use of available data
	Commitments may indicate future trends		
Weaknesses	Commitments are not always met, so do not necessarily reflect the actual value made available to recipients	Relatively complete data only available from 2002	Difficult to interpret if combined: the proportion of commitments that result in disbursements and the delay from commitment to disbursement varies between donors and types of projects and over time
	Changes difficult to interpret especially annually at donor or recipient level because they are “lumpy”: the entire value of a multi-year project is reported as a commitment in a single year		

**Table 3:** Examples of the three main analytical approaches and two possible measures for analyzing health aid to specific areas.

Analytical Approach	Measure		
	Commitment	Disbursement	Both
Existing CRS sector and purpose codes	Malaria (Snow et al., 2010) [49] a	MNCH (G8, 2010) [26]	HIV/AIDS, health sector support, and other areas (Piva and Dodd, 2009) [50] b
		Malaria (Akachi et al., 2011) [51]	
		UK aid for human resources for health (Campbell et al., 2011) [25] a	
		Reproductive health in conflict-affected countries (Patel et al., 2009) [24]	
Key term searches in titles and descriptions	(No examples)	Mental health and psychosocial support in humanitarian settings (Tol et al., 2011) [52] c	HIV/AIDS, tuberculosis, malaria, health sector support (IHME/Ravishankar et al., 2009) [53]
			MNCH, malaria, HIV, health systems, tuberculosis, non-communicable diseases, and health sector support (IHME, 2010) [17] d
Coding of individual records based on a predefined framework	Neglected tropical diseases (Liese et al., 2009) [54]	MNCH (Countdown/Pitt et al., 2010) [12] a	MNCH (Countdown/Powell-Jackson et al., 2006) [14] a ^,^ e
		20 communicable diseases (Shiffman, 2006) [55]	MNCH (Countdown/Greco et al., 2008) [15] a,e

a Additional data sources used to complement the CRS.

b Analyses based on commitments; a limited comparison of disbursement and commitment data conducted.

c Not clearly specified whether disbursement or commitment data used, but disbursements are suggested.

d Commitments and disbursements used to estimate disbursements for analyses.

e Analysis based on disbursement data except for the World Bank, which only reported commitments.

The different analytical approaches have attributed either the entire value of the commitment or disbursement to their focus area, or only a portion thereof. These proportions have been derived in various ways from assumption [13],[17], through review of existing data and literature [14], to in-depth case studies [25]. For example, the G8 used the existing purpose codes to attribute a proportion of the value of each disbursement to MNCH, and based these percentages largely on assumptions [26]. The IHME characterized all development assistance for health, and so produced its own database of estimated disbursements largely based on the commitment and disbursement data in the CRS, and then used key term searches of records within the CRS's health and population sector codes to categorise disbursements as related to MNCH, HIV, tuberculosis, malaria, non-communicable diseases, health systems, or “other” [13],[17]. IHME attributed the entire value of the disbursement to one of the five categories unless key terms for multiple categories were found in the same record, in which case the value of the disbursement was divided evenly across the categories, or unless the descriptions did not include any of their search terms, in which case the value of the disbursement was attributed to “other” [13],[17]. The Countdown to 2015 individually recoded each record on the basis of a predefined framework, and then for each of these new codes, attributed between 0% and 100% of the value of the disbursement to MNCH, with the particular percentages for each code determined by a review of the literature and existing data on, for example, the proportion of general government expenditure spent on health [12],[14],[15].

## Methods to Analyse Donor Funding for Newborns

We sought to analyse donor funding for newborn health in such a way as to maximize the sensitivity and specificity of our findings and to generate transparent and reproducible results that would consequently be accepted by donors and promote accountability. We therefore carefully developed an automated key term approach and then applied this key term approach to disbursements in both the most recent version of the CRS database and the Countdown database. We reviewed records individually both to assist in developing the search strategy, and to explore the findings further.

### Step 1: Development of Search Terms

To develop the key terms, we began by defining our target records as those mentioning newborns or whose descriptions indicated that they supported interventions in pregnancy or in the first 4 wk of life that are proven to improve or maintain newborn health and/or reduce stillbirths or miscarriage. A range of scientific literature [7],[27]–[30] was consulted to identify an initial set of search terms in English within three categories: general newborn terms, newborn conditions and diseases, and newborn interventions and programmes. While the CRS directives instruct donors to report in English or French [21], donor records include German, Dutch, Spanish, Portuguese, and Italian text. All search terms were therefore translated into each of these languages to maximize sensitivity of results and to limit bias. Search terms were truncated as much as possible without compromising specificity. Search terms were not case-sensitive. Diseases, conditions, interventions, and programmes that invariably include general terms such as “newborn” or “neonatal” (e.g., “neonatal resuscitation”) were not included separately, as they would be captured by the general term.

### Step 2: Refinement of Search Methods

The initial search term list was piloted in the Countdown database, which covered the years 2003–2008, and all records identified were individually reviewed. There were 110 search terms that did not locate any project records (Text S1). The 28 search terms that identified one or more projects were refined where possible to maximize specificity without compromising sensitivity. For example, the initial search term “n?onat” including the single wildcard character “?” was replaced with two terms, “neonat” and “néonat” to identify the English words “neonate,” “neonates,” “neonatal,” and “neonatology”, as well as the corresponding Dutch, German, Italian, and French terms, while ceasing to identify, for example, projects with the German word “Stipendienmonaten” (“monthly grant payment”) or mentioning the town of Sonsonate in El Salvador. In a number of cases, a single space was added to the beginning of search terms, changing, for example, “fetal” to “_fetal” to avoid misclassifying records containing unrelated Spanish words such as “cafetaleros” (“coffee growers”), as doing so was not found to compromise sensitivity.

Six search terms consistently identified projects unrelated to newborns without adding any newborn activities to those already identified by other search terms; they were therefore removed from the final search term list (Table 4). For example, the search term “tetanus” was removed, as it frequently identified projects providing DPT3, tetravalent, or pentavalent vaccines to older children, while projects focussed on maternal and neonatal tetanus were already identified by other search terms, notably “neonat” and “toxoid.” The abbreviation “KMC,” which we used to look for funding for kangaroo mother care, only identified projects supporting Kunming Medical College, and so was also removed. The final list of 24 search terms that effectively identified newborn aid activities are listed in Table 5.

**Table 4:** Search terms consistently misclassifying disbursements without identifying additional newborn disbursements.

Search Term	Explanation
pr?matur	All relevant projects identified by other search terms. Frequently identified the French word “primature” (“office of the prime minister”).
KMC	Misclassified projects referring to “Kunming Medical Centre.” Never found referring to “Kangaroo Mother Care.”
_SP_	All relevant projects identified by other search terms. Did not add projects related to intermittent preventive treatment of malaria in pregnancy as intended.
st?ro?d	All relevant projects identified by other search terms. Misclassified projects with the words “ministerio della,” “forest roads,” and “west road.”
_cord?n	Did not add projects related to care of the umbilical cord.
tetanus	All relevant projects identified by other search terms. Often found in reference to DPT, tetravalent, and pentavalent vaccines for children, rather than maternal or neonatal tetanus immunization.

**Table 5:** Final list of search terms identifying projects that benefit newborns ranked by number of additional records identified.

Search Term (Translation, Language)	Value of Projects Identified	Value of Additional Projects Identified (beyond Those Already Identified by Search Terms Higher on the List)
Total	4,538 (100.0%)	4,538 (100.0%)
newborn	2,913 (64.2%)	2,913 (64.2%)
breastfe	698 (15.4%)	657 (14.5%)
neonat	630 (13.9%)	558 (12.3%)
_p?rinat	169 (3.7%)	126 (2.8%)
birth?weight	89 (2.0%)	45 (1.0%)
_postnat	71 (1.6%)	45 (1.0%)
malaria in pregnancy	41 (0.9%)	39 (0.9%)
IPTp	22 (0.5%)	22 (0.5%)
MNH	31 (0.7%)	21 (0.5%)
nouveau-n (newborn, French)	64 (1.4%)	17 (0.4%)
syphilis	19 (0.4%)	16 (0.4%)
reci?n nacido (newborn, Spanish)	31 (0.7%)	15 (0.3%)
néonat	22 (0.5%)	13 (0.3%)
borstvoed (breastfeed, Dutch)	13 (0.3%)	13 (0.3%)
MNCH	26 (0.6%)	11 (0.2%)
n?o-nat	13 (0.3%)	7 (0.2%)
s?filis (syphilis, Spanish)	9 (0.2%)	6 (0.1%)
toxoid	8 (0.2%)	4 (0.1%)
_fetal	8 (0.2%)	4 (0.1%)
breast-fe	5 (0.1%)	3 (0.1%)
_fetus	3 (0.1%)	2 (0.0%)
stillb	2 (0.0%)	1 (0.0%)
cord care	43 (0.9%)	0 (0.0%)
Kangaro	1 (0.0%)	0 (0.0%)

?, single character wildcard.

_, used to highlight the presence of a blank character.

### Step 3: Application of Search Terms to CRS and Countdown Databases

We applied the final set of newborn search terms to all sectors of the CRS database except for action relating to debt (i.e., debt forgiveness) for the years from 2002, the first year for which relatively complete disbursement data are available, to the most recent year available, 2010, using Visual Basic for Applications macros in Excel 2007 (Microsoft). The December 2011 update of the CRS database was used for 2002–2009 data, while the April 2012 update was used for 2010 data. In addition to positive and negative ODA grant disbursements and positive ODA loan disbursements, we also included private grants from the Bill & Melinda Gates Foundation, which began reporting its disbursements from 2009 and has been recognised as an important actor in global health [31]. We included all recipients and all donors in the CRS. All records with a missing or zero disbursement value were excluded, as were loan repayments (i.e., negative values for ODA loans). While we are interested in newborn health, we searched in non-health sectors to capture both multipurpose projects and programmes whose main purpose was deemed to fall outside of the health sector, as well as those that were miscoded by donors.

We also applied the final set of search terms to the Countdown database to generate results that could be compared directly with the Countdown estimates of aid to MNCH.

### Step 4: Individual Identification of Misclassified Records, Records Exclusively Benefitting Newborns, and Research Funding

All projects identified by the final list of newborn search terms within each database were individually reviewed and classified according to whether the funded activities (1) aimed to benefit newborns exclusively, (2) aimed to improve the health of other population groups as well as newborns, or (3) were misclassified because the search term identified a record wholly unrelated to newborn health. Misclassified projects were removed from subsequent analysis. Separately, all correctly identified records in the CRS database were categorised as to whether they funded research or non-research activities. CP and MR independently coded the final set of records identified by the search of the CRS database, 2002–2010 and agreed a final set of codes.

### Step 5: Analysis

We considered our primary analysis to be that of the CRS database for 2002–2010. We present the total number and value of records identified by our search terms, as well as the number and value of records aiming exclusively to benefit newborns. We do not apportion a share of the value of records mentioning newborns alongside other population groups, but instead compare the full value of all records identified by our search terms to the value of projects exclusively benefitting newborns and wider estimates of funding to maternal and newborn health (MNH), MNCH, the health sector as a whole, and the health and humanitarian sectors combined. Results were disaggregated by year, donor, and recipient country to examine patterns and trends over time. Results were compared between the Countdown and CRS databases, and also with the IHME key term approach (Text S1) and estimates of funding to MNCH [13],[17]. Finally, we generate estimates of aid mentioning newborns and exclusively benefitting newborns per live birth [12],[32]. Values are presented in constant 2010 US$.

## Results

### Methodological Findings

In total, 4,584 grant or positive loan disbursement records within the selected sectors of the CRS database contained at least one newborn search term for the years 2002 to 2010. Independent coding of all 4,584 records resulted in high levels of agreement (98.4%, κ = 0.91) regarding whether records exclusively benefitted newborns, also benefitted other population groups, or were misclassified, and moderate levels of agreement (97.3%, κ = 0.47) regarding whether correctly classified projects supported research or non-research programmatic and advocacy activities (Text S1). Reported data are based on agreed codes.

Of the 4,584 identified records, 46 were misclassified and have been excluded (Text S1). They represented 1.0% of the number of records with newborn terms in the CRS database and 1.7% of their value. The search strategy was therefore highly specific, but individual review and removal of these misclassified records avoided a potential bias as four-fifths were of a single donor, Germany, which happened to include a reference code within its description of food aid projects that included the letters “MNH.”

Each of the 4,538 correctly classified records was identified by a minimum of one and a maximum of eight different search terms. Search terms were ranked according to the number and value of additional project records they identified (Tables 5–6). Greater than 78% of identified records were identified by the search terms “newborn” or “neonat.” The search term “breastfe” was found in 41 projects already identified by the search term “newborn,” but also identified an additional 657 records without the term “newborn” (Table 5), of which just 37 would have been identified by subsequent search terms. Several search terms, such as “cord care” did not misclassify records, but also did not identify any additional records and were therefore superfluous. The search term “stillb” and its translations, which aimed to capture references to stillborns or stillbirths, identified only two projects, both in 2010: funding from the Gates Foundation for The Lancet Stillbirth Series, and funding from Switzerland to Tanzania to equip 45 health facilities with equipment for emergency obstetric and newborn care. All the search terms aiming to capture references to a “fetus” or “fetal” (including spelling variants) correctly identified a total of nine records valued at US$15.5 million between 2002 and 2010, of which seven provided US$14.9 million for research (Table 5; Text S1).

**Table 6:** Final list of search terms identifying projects that benefit newborns ranked by value of additional records identified.

Search Term (Translation, Language)	Value of Projects Identified	Value of Additional Projects Identified (beyond Those Already Identified by Search Terms Higher on the List)
Total	2,551.4 (100.0%)	2,551.4 (100.0%)
newborn	1,394.6 (54.7%)	1,394.6 (54.7%)
neonat	653.4 (25.6%)	552.9 (21.7%)
breastfe	298.3 (11.7%)	288.3 (11.3%)
malaria in pregnancy	95.5 (3.7%)	95.2 (3.7%)
_p?rinat	125.2 (4.9%)	78.2 (3.1%)
IPTp	43.6 (1.7%)	43.6 (1.7%)
MNCH	65.4 (2.6%)	26.7 (1.0%)
birth?weight	60.4 (2.4%)	21.0 (0.8%)
syphilis	24.4 (1.0%)	19.1 (0.8%)
_postnat	154.9 (6.1%)	7.4 (0.3%)
reci?n nacido (newborn, Spanish)	13.2 (0.5%)	4.9 (0.2%)
néonat	8.2 (0.3%)	5.4 (0.2%)
borstvoed (breastfeed, Dutch)	5.0 (0.2%)	5.0 (0.2%)
n?o-nat	48.7 (1.9%)	2.6 (0.1%)
nouveau-n (newborn, French)	7.4 (0.3%)	2.5 (0.1%)
_fetus	6.3 (0.2%)	1.0 (0.0%)
_fetal	15.2 (0.6%)	0.9 (0.0%)
Toxoid	4.7 (0.2%)	0.8 (0.0%)
breast-fe	2.3 (0.1%)	0.5 (0.0%)
s?filis (syphilis, Spanish)	0.5 (0.0%)	0.4 (0.0%)
MNH	22.7 (0.9%)	0.2 (0.0%)
stillb	2.3 (0.1%)	0.2 (0.0%)
cord care	14.5 (0.6%)	0.0 (0.0%)
Kangaro	0.7 (0.0%)	0.0 (0.0%)

?, single character wildcard.

_, used to highlight the presence of a blank character.

The vast majority of records mentioning newborns had been classified by donors as falling within the population and reproductive health sector (sector code 130, 67.5% by number, 56.3% by value), or the health sector (sector code 120, 29.2% by number, 38.9% by value), while less than 1% of records were found in the emergency response sector (sector code 720, 0.8% by number, 0.1% by value). However, 4.1% of the value of records mentioning newborns were found in other sectors over the 9-y period and over 17% were found in other sectors in 2003–2005, reflecting a combination of multi-sector interventions and those clearly misclassified by donors (Text S1).

The search strategy was more sensitive than that employed by IHME [13],[17] to identify funding to MNCH. After removing accents from our set of newborn records, we found that the IHME search terms (Text S1) would have identified 82.4% (by number) or 82.6% (by value) of the correctly classified newborn records we identified. The IHME search terms would have identified an even smaller proportion of records exclusively benefitting newborns (21.5% by number, 78.5% by value). Projects with titles such as “newborn care,” “neonatology,” and “breastfeeding” were not identified by the IHME search terms.

**Figure 1:**
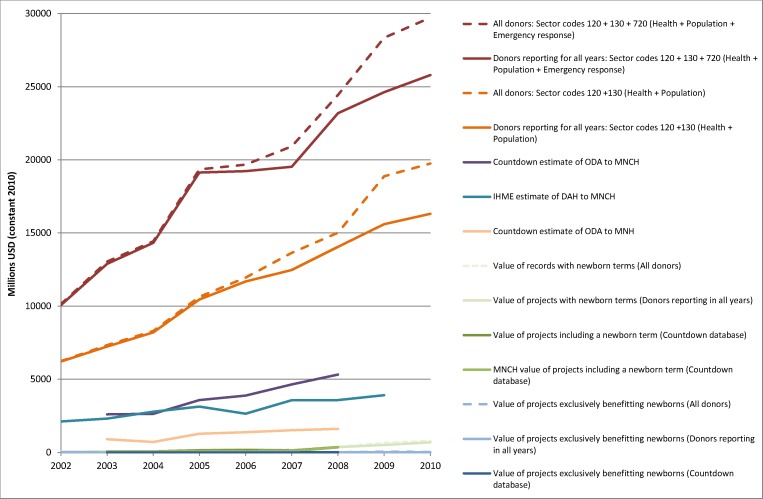
Estimates for the value of aid benefitting newborns in context. The figure shows several options for estimating how much aid is benefitting newborns in the context of overall aid to the health and emergency response sectors. As a lower bound of estimation of the value of aid benefitting newborns, the dashed and solid blue lines reflect the value of projects exclusively benefitting newborns. The green lines reflect the value of aid disbursements whose descriptions mention a newborn search term in each of the two databases. As upper bounds of estimation of the value of aid benefitting newborns, the solid peach, turquoise, and purple lines reflect estimates of the total value of aid for MNH or for MNCH. The orange and dark red lines indicate the value of aid to the health and population sectors as a whole, with the dark red also including humanitarian aid. Solid lines reflect estimates based on donors who reported in all years, while dashed lines reflect estimates based on all available data, including donors who may not have reported in all years. DAH, development assistance for health.

**Figure 2:**
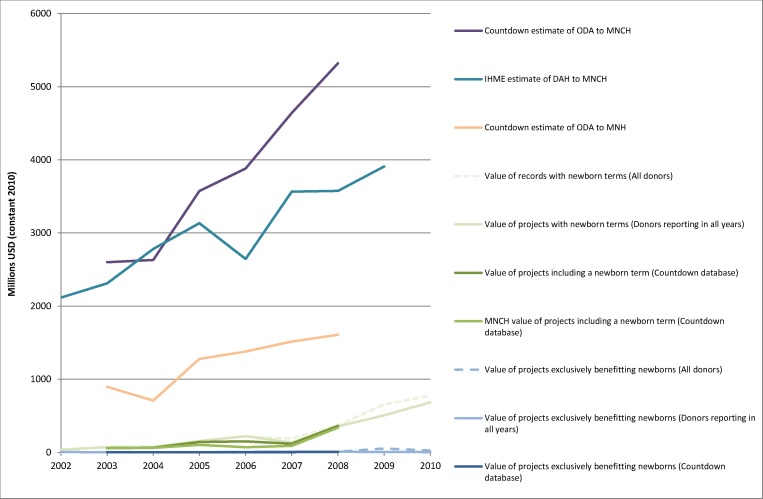
Estimates for the value of aid benefitting newborns in context. The figure presents the same data as in Figure 1 except in that it excludes the estimates of aid to the health, population, and humanitarian sectors as a whole, and is on a 5-fold smaller scale to enable closer examination of estimates specific to newborns and their relationship to estimates of aid for MNH and MNCH. DAH, development assistance for health.

**Figure 3:**
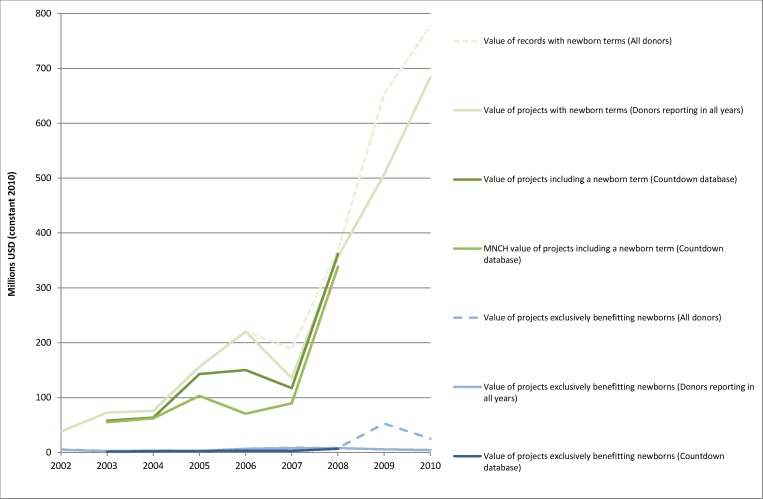
Estimates of the value of aid including newborn search terms and exclusively benefitting newborns. The figure presents the same data as in Figure 2 except in that it excludes the estimates of aid for MNCH and MNH, and is on a 7.5-fold smaller scale to enable closer examination of estimates specific to newborns.

When our search strategy was repeated within the Countdown database, the results were broadly similar (Figures 1–3), although results from the Countdown database were slightly lower in each of the 6 y included in the database. Detailed comparison of the findings revealed that this difference was primarily caused by changes donors made to their reported data after it had been included and analysed in the Countdown database. However, some records identified in the CRS had also been excluded from the Countdown database either because they were research projects or because the purpose code and two of the three text fields indicated that they were not contributing to MNCH. The World Bank, for example, separates each of its many multi-sector projects into many different records with different purpose codes and different disbursement values, but repeats the same description of all the sectors of activity; thus, while the Countdown database only includes the disbursements relevant to health, the newborn key term search identifies every record with the newborn term, including those disbursements that are supporting activities in another sector.

### Data Findings Relevant to Newborns

**Figure 4:**
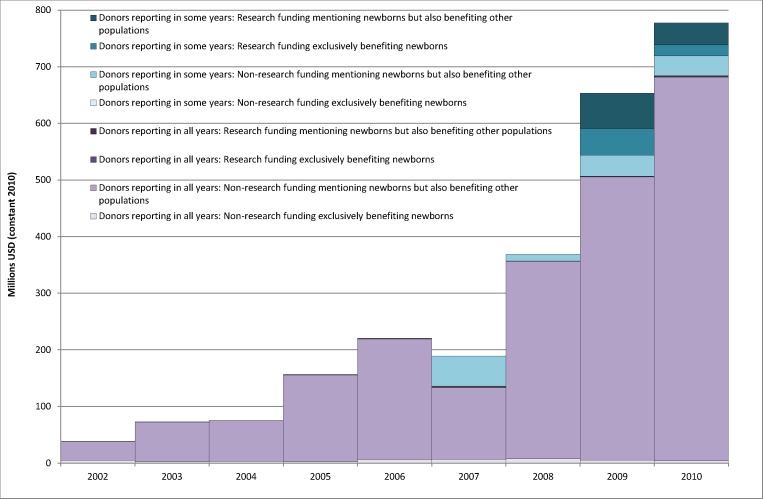
The value of records mentioning newborn search terms, 2002–2010. The figure presents the value of aid identified by the search strategy in constant 2010 US$. Data are disaggregated by (1) whether the donor reported to the CRS in all 9 y (shown in purple) or in fewer than 9 y (shown in turquoise); (2) whether the funding exclusively benefits newborns or also benefits other population groups; and (3) whether the funding supported research or non-research (i.e., programme or project implementation or advocacy) activities. The figure demonstrates that a large majority of aid including a newborn search term was provided for non-research activities that also benefitted other population groups by donors who reported in all years. In 2009 and 2010, the Gates Foundation, which only reported in those years, provided significant funds for research. The following donors (shown in turquoise) mentioned newborns in at least one disbursement but reported for only some of the 9 y: The Bill & Melinda Gates Foundation (2009–2010), Denmark (2003–2010), Finland (2002–2003, 2006–2010), the GAVI Alliance (2007–2010), Korea (2006–2010), the Organization of the Petroleum Exporting Countries' Fund for International Development (OFID, 2009–2010), and the World Health Organization (2009–2010). The Global Fund reported for 2003–2010, but has been included amongst donors who reported for all years as their publicly available data indicate that their total disbursements in 2002 constituted less than US$1 million and was provided to Ghana for HIV and tuberculosis.

The total value of records identified by a newborn search term rose from US$38.4 million in 2002 to US$777.3 million in 2010 (constant 2010 US$). As illustrated in Figure 4, some donors, most notably the Gates Foundation and the GAVI Alliance, did not report in all years, and so their inclusion somewhat inflates the upward trend. Nonetheless, even considering only donors who reported in all 9 y, the value of aid mentioning newborns rose from US$38.1 million in 2002 to US$683.8 million in 2010. The vast majority of this aid supported programmatic or advocacy (i.e., not research) activities that mentioned newborns but also benefitted other populations. The value of non-research activities exclusively benefitting newborns was US$5.1 million in 2002, fell as low as US$2.4 million in 2003 and 2005, rose as high as US$7.6 million in 2008, and in 2010 was US$5.7 million. Including only donors who reported in all years results in similar fluctuations, but an overall drop in non-research funding exclusively benefitting newborns, from US$4.8 million in 2002 to US$4.2 million in 2010. Funding of projects exclusively benefitting newborns was therefore very low, and represented a relatively small proportion (4.4% by value, 10.0% by number) of records including a newborn search term.

Amongst donors who reported in all years, very little research funding mentioning newborns was identified: from US$75,000 in total for 2002–2004 combined, their research contributions rose to just over US$2.0 million in 2010. By contrast, in the 2 y in which it reported, 2009 and 2010, the Gates Foundation provided US$109.0 million and US$58.2 million in research funding, respectively, of which US$46.8 million (42.9%) and US$19.4 million (33.4%) exclusively benefitted newborns. Research funding was thus a small proportion of overall funding (6.9%), even in 2010 (7.7%), but constituted a much larger proportion (77.2%) of the value of aid exclusively benefitting newborns in 2010.

Compared with either the Countdown or IHME's [16],[17] estimates of funding to MNCH, or even compared with the Countdown's estimate of funding only to MNH, the value of records including a newborn search term is very low (Figures 1–2). These figures are in turn dwarfed by the overall value of aid to health, population, and humanitarian sectors as a whole (Figure 1). From 2003 to 2008, the period for which Countdown estimates are available, the proportion of aid for MNCH that mentioned newborns rose from 2.1% to 6.3%. Within the Countdown database, the total disbursement value and the value attributed to MNCH for records including a newborn search term are very similar (Figures 1–2) because nearly all records identified by the newborn search terms were entirely focused on one or more aspects of MNCH. Only 6.4% of records mentioning newborn search terms described primary health care, generic hospital, or health systems projects or sector support for which less than the full value of the disbursement would be counted towards MNCH in the Countdown framework.

Of the 45 bilateral donors (*n* = 25), multilateral donors (*n* = 17), global health initiatives (*n* = 2), and private foundations (*n* = 1) that reported health or population sector disbursements to the CRS database, 32 financed at least one project including a newborn search term in the descriptive text between 2002 and 2010. The number of donors including a newborn search term in at least one of their project descriptions rose from 15 in 2002 to 25 in 2010 and from 14 to 21 amongst donors who reported in all years, but this rise was not consistent, as donors mentioning newborns in a funding description one year did not always continue to mention newborns in funding descriptions in subsequent years.

The United States was by far the largest contributor to activities identified by the newborn search terms, providing US$1114.8 million in aid mentioning newborns, of which 99.5% supported activities also benefitting other population groups. The next highest contributors were The World Bank (US$413.4 million), and Canada (US$208.5 million), neither of which provided any funding exclusively benefitting newborns, and the Gates Foundation (US$232.9 million) (Table 7). Despite reporting in only 2 of the 9 y, the Gates Foundation accounted for 60.3% of all funding exclusively benefitting newborns, 97.7% of all research funding exclusively benefiting newborns, and 94.1% of the research funding supporting both newborns and other population groups. We classified 71.8% of the value of the Gates Foundation's funding for newborns as supporting research, while the remaining non-research activities generally supported advocacy, policy change, and efforts to increase coverage of existing interventions. UNICEF was the largest contributor of non-research funding exclusively benefitting newborns, providing generally small disbursements to 94 countries for projects described simply as “newborn care” or “newborn care in the community.”

**Table 7:** Total value of aid for newborns by category and donor, 2002–2010.

Donor	Mentions Newborns but also Benefits Other Population Groups		Exclusively Benefits Newborns		Total
	Research	Not Research	Research	Not Research	
Bilateral	5.9	1,613.7	1.6	31.8	1,653.0
Australia	0.0	82.9	0.0	1.0	83.9
Austria	0.0	0.1	0.0	0.0	0.1
Belgium	1.4	4.2	0.0	1.9	7.5
Canada	0.0	208.5	0.0	0.0	208.6
Denmark^a^	0.0	0.1	0.0	0.0	0.1
Finland	0.0	0.1	0.0	0.8	0.9
France	0.2	2.0	0.0	0.1	2.3
Germany	0.0	13.4	0.0	0.0	13.4
Greece	0.0	0.0	0.0	0.2	0.2
Ireland	0.2	0.2	0.0	0.0	0.3
Italy	0.0	1.5	0.0	0.5	2.0
Japan	0.0	5.1	0.0	6.6	11.6
Korea	0.0	1.7	0.0	0.5	2.1
Luxembourg	0.0	2.8	0.0	0.0	2.8
Netherlands	0.0	24.6	0.0	0.0	24.6
New Zealand	0.0	1.7	0.0	0.0	1.7
Norway	0.4	38.3	0.0	0.5	39.2
Portugal	0.0	0.2	0.0	0.0	0.2
Spain	0.2	39.2	0.0	2.2	41.5
Sweden	1.4	3.9	0.0	0.0	5.4
Switzerland	0.0	5.7	0.0	8.3	14.0
United Kingdom	1.4	68.6	1.6	4.0	75.7
United States	0.7	1,109.0	0.0	5.2	1,114.8
Global	0.0	164.3	0.0	0.0	164.3
GAVI^b^	0.0	63.7	0.0	0.0	63.7
Global Fund	0.0	100.6	0.0	0.0	100.6
Multilateral	0.4	488.4	0.0	12.4	501.2
EU Institutions	0.0	17.5	0.0	0.0	17.5
OFID^c^	0.0	0.1	0.0	0.0	0.1
UNFPA	0.0	4.7	0.0	0.0	4.7
UNICEF	0.0	46.5	0.0	12.4	58.9
WHO^c^	0.4	6.2	0.0	0.0	6.6
World Bank (IDA)	0.0	413.4	0.0	0.0	413.4
Private	101.0	64.1	66.2	1.6	232.9
Bill & Melinda Gates Foundation^c^	101.0	64.1	66.2	1.6	232.9
Grand total	107.3	2,330.5	67.8	45.8	2,551.4

The following donors did not mention any newborn search terms although they did report disbursements to the health sector in one or more years, 2002–2010: Kuwait, United Arab Emirates, African Development Fund, Arab Fund for Economic and Social Development, Asian Development Bank Special Fund, Global Environment Facility, International Development Bank Special Fund, UNAIDS, United Nations Development Program, United Nations Economic Commission for Europe, United Nations Peacebuilding Fund, United Nations Relief and Works Agency, World Food Program.

a Reported 2003–2010 only.

b Reported 2007–2010 only.

c Reported 2009–2010 only.

Nearly a quarter of aid mentioning newborns (23.2%, US$595.6 million) was not allocated to specific recipient countries, but rather classified as supporting regional (US$182.4 million) or “bilateral, unspecified” recipients (US$414.5 million) (Table 8). Three donors provided 91.9% of the “unspecified” funding: the United States (US$209.5 million), whose “unspecified” funding supported a range of non-governmental organisations, private consultancies, research organisations, and multilateral organisations; the Gates Foundation (2009 and 2010 data only: US$107.9 million), which provided US$23.4 million for the Saving Newborn Lives programme (which supported this research) and the remainder to unnamed organisations to carry out specific projects; and GAVI (US$63.7m), whose “unspecified” aid entirely reflected its contributions to the Maternal and Neonatal Tetanus Elimination Initiative.

**Table 8:** The leading regional and unspecified recipients of total aid mentioning and exclusively benefitting newborns over the period 2002–2010 and in 2010 (constant 2010 US$, millions).

Rank	Mentioning Newborns, 2002–2010	Mentioning Newborns, 2010	Exclusively Benefitting Newborns, 2002–2010	Exclusively Benefitting Newborns, 2010
1	“Bilateral, unspecified” (US$414.49)	“Bilateral, unspecified” (US$139.73)	“Bilateral, unspecified” (US$50.42)	“Bilateral, unspecified” (US$9.79)
2	“South of Sahara, regional” (US$73.6)	“South of Sahara, regional” (US$11.99)	“South of Sahara, regional” (US$0.73)	“Oceania, regional” (US$0.02)
3	“Africa, regional” (US$68.37)	“Africa, regional” (US$3.16)	“America, regional” (US$0.06)	—
4	“America, regional” (US$12.46)	“America, regional” (US$2.42)	“Oceania, regional” (US$0.03)	—
5	“North & Central America, regional” (US$10.71)	“Asia, regional” (US$1.29)	—	—
6	“Asia, regional” (US$7.91)	“Central Asia, regional” (US$0.19)	—	—
7	“Central Asia, regional” (US$0.63)	“Oceania, regional” (US$0.04)	—	—
8	“South & Central Asia, regional” (US$0.33)	“Europe, regional” (US$0)	—	—
9	“Europe, regional” (US$0.31)	—	—	—
10	“Oceania, regional” (US$0.12)	—	—	—

Recipients defined in the CRS directives and used in the CRS database [21].

The value of records containing a newborn search term varied considerably between recipient countries both in total and relative to the number of births in each country (Tables 9–10). The four countries receiving the greatest funding to include a newborn search term over the period, Bangladesh (US$322.6 million), Afghanistan (US$156.9 million), Pakistan (US$150.1 million), and India (US$134.3 million) (Table 9), are all in South Asia and amongst the countries with the highest numbers and rates of neonatal deaths [3]. Yet, middle-income countries [33] with small populations tended to lead the lists of countries receiving the largest value of aid mentioning newborns and exclusively benefitting newborns per live birth (Table 10). In 2010, Samoa received the largest value of aid mentioning newborns per live birth with US$287.0 for each of its estimated 4,260 live births [32] from a World Bank (IDA) loan of US$1.2 million for a health sector management programme, which cited neonatal mortality reduction amongst its many objectives. Zambia received US$2.1 million in 2010 for a Gates-funded efficacy trial of chlorhexidine umbilical cord cleaning, which made it the largest recipient of aid per birth exclusively benefitting newborns that year. For individual recipient countries, aid also varied significantly from year to year. In some years, some recipients even seemed to experience negative disbursements, reflecting a net return of monies to the donor.

**Table 9:** The leading country recipients of total aid mentioning and exclusively benefitting newborns over the period 2002–2010 and in 2010 (constant 2010 US$, millions).

Rank	Mentioning Newborns, 2002–2010	Mentioning Newborns, 2010	Exclusively Benefitting Newborns, 2002–2010	Exclusively Benefitting Newborns, 2010
1	Bangladesh (US$322.63)	Afghanistan (US$72.31)	India (US$10.42)	Zambia (US$2.13)
2	Afghanistan (US$156.88)	Pakistan (US$53.81)	Moldova (US$5.88)	Tanzania (US$1.66)
3	Pakistan (US$150.08)	India (US$53.4)	Pakistan (US$5.8)	India (US$1.01)
4	India (US$134.32)	Bangladesh (US$41.91)	Zambia (US$5.27)	Pakistan (US$0.34)
5	Tanzania (US$108.05)	Nigeria (US$31.37)	Tanzania (US$3.74)	Egypt (US$0.22)
6	Indonesia (US$63.34)	Indonesia (US$30.85)	Ukraine (US$2.53)	Ghana (US$0.22)
7	Nigeria (US$62.06)	Ethiopia (US$24.4)	Mozambique (US$1.61)	Nigeria (US$0.19)
8	Sudan (US$50.91)	Sudan (US$18.77)	Nepal (US$1.58)	Bolivia (US$0.17)
9	Nicaragua (US$50.70)	Cambodia (US$16.14)	Uzbekistan (US$1.53)	Uzbekistan (US$0.17)
10	Ethiopia (US$49.34)	Jordan (US$15.23)	Palestinian Administrative Areas (US$1.37)	Rwanda (US$0.17)

**Table 10:** The leading country recipients of aid per live birth mentioning and exclusively benefitting newborns over the period 2002–2010 and in 2010 (constant 2010 US$).

Rank	Mentioning Newborns, 2002–2010	Mentioning Newborns, 2010	Exclusively Benefitting Newborns, 2002–2010	Exclusively Benefitting Newborns, 2010
1	Nicaragua (US$28.14)	Samoa (US$286.96)	Moldova (US$10.26)	Zambia (US$3.72)
2	Samoa (US$26.09)	Jordan (US$90.53)	Macedonia, FYR (US$1.21)	Timor-Leste (US$1.13)
3	Georgia (US$20.87)	Georgia (US$63.08)	Zambia (US$0.80)	Uruguay (US$1.01)
4	Eritrea (US$20.73)	Liberia (US$54.82)	Palestinian Administrative Areas (US$0.73)	Tanzania (US$0.89)
5	Jordan (US$16.31)	Afghanistan (US$53.29)	Uruguay (US$0.49)	Nicaragua (US$0.87)
6	Armenia (US$12.78)	Armenia (US$44.74)	Ukraine (US$0.44)	Maldives (US$0.76)
7	Moldova (US$11.16)	Cambodia (US$43.07)	Papua New Guinea (US$0.40)	Bolivia (US$0.62)
8	Afghanistan (US$10.48)	Haiti (US$40.00)	Eritrea (US$0.31)	Rwanda (US$0.40)
9	Liberia (US$8.97)	Timor-Leste (US$34.59)	Uzbekistan (US$0.22)	Tajikistan (US$0.33)
10	Haiti (US$8.58)	Burundi (US$30.01)	Honduras (US$0.19)	Uzbekistan (US$0.30)

## Discussion

We found that the value of aid disbursements mentioning newborns or an activity expected to benefit newborns rose 20-fold between 2002 and 2010 and constituted an increasing proportion of aid to MNCH and to the health sector as a whole. While the increase in mentions of newborns within wider projects and programmes may in part simply reflect increased detail in overall reporting, when contrasted against stillbirths, which were mentioned in only two projects, and the fetus, which was mentioned in only nine disbursements during the 9-y period, it becomes clear that newborns have indeed received increased attention. These findings support those of a qualitative study charting the rise of newborn health as a global health issue [11]. Most of this rise we document occurred after 2005, when *The Lancet* Neonatal series published the first estimates of neonatal cause of death by country and a cost-effectiveness analysis of interventions, including those possible at community level, which helped to mobilise wider attention for newborn survival [11]. Our findings also support the conclusions of more recent editorials and epidemiological studies highlighting the lack of attention for stillbirths on the global health agenda, despite their public health importance [34]–[40].

At just US$5.7 million in 2010, the value of non-research projects exclusively benefitting newborns was very low; however, firm estimates of the total value of aid benefitting newborns remain elusive. Domestic expenditure is a far greater proportion of total health expenditure in most developing countries, and so no analysis of donor funding can provide a complete estimate of the resources available for newborn health. Furthermore, while the Countdown Initiative includes a share of broader funding for health systems and primary and secondary care in its estimates of funding for MNCH, neither this analysis nor that of IHME takes into account such funding unless the record explicitly includes a newborn search term. Thus, while our results provide relatively convincing indications of a positive trend in donor attention for newborns, they cannot provide a comprehensive estimate of the value of resources or even the value of donor aid benefitting newborns in developing countries.

The vast majority of aid activities identified by the newborn search terms aimed to benefit mothers and/or children older than 1 mo of age, as well as mentioning newborns. As interventions for newborns are most likely to be cost-effective when integrated into a package of activities benefitting mothers and older children [7], and a newborn's health is dependent on her mother's health [41], this integration, especially in programmatic funding, is likely positive. Nonetheless, analyses such as this one based only on funding descriptions cannot determine whether the increased inclusion of newborn search terms in these wider projects reflects a genuine shift in activities, or a superficial change in funding titles and descriptions, for example, from “maternal and child health” to “maternal, newborn, and child health.”

Recipient countries and implementing partners also have wide latitude to determine how much MNCH funding from donors benefits newborns in practice. At the negative extreme, they may focus all MNCH resources on activities of benefit only to mothers and older children, even where newborns are mentioned in the funding description. At the positive extreme, they may use funding whose description does not explicitly mention newborns to achieve positive synergies by ensuring that, for example, skilled birth attendants not only look after the mother but also prevent stillbirths by monitoring the fetal heart rate and reduce neonatal deaths by performing neonatal resuscitation when necessary [42],[43]. The actual amount of aid benefitting newborns in any given country could therefore conceivably be as low as the value of projects exclusively benefitting newborns, or potentially as high as a major proportion of the total value of the Countdown's estimates of ODA to MNCH, if the potential for synergies and double-impact on both the mother and fetus or newborn are taken advantage of and taken into account (Figure 2).

For 2010, we identified US$19.4 million in aid for research projects exclusively benefitting newborns. While we initially distinguished between research and non-research funding to permit direct comparisons with the Countdown's estimates of aid to MNCH, which excluded research funding, the degree to which research funding exclusively benefitting newborns (US$67.8 million) outweighed programmatic funding exclusively benefitting newborns (US$45.8 million) over our 9-y period of analysis is striking. That the Gates Foundation provided 97.7% of this research funding exclusively for newborns while only reporting in the final two of the 9 y is all the more remarkable, but also means that we cannot draw any conclusions regarding trends over time in research funding for newborns. Further research is important, especially into effective ways to increase coverage of key interventions [44],[45]; however, while some research participants may receive direct benefits, most of the benefits of research are not reaped for several years or more. As newborns now account for over 40% of child deaths and strong evidence is already available on the efficacy of a range of interventions that are expected to be highly cost-effective [7], an expansion of programmatic funding is urgently needed to promote efficient and equitable implementation of these proven newborn survival interventions.

Our analysis has wider value as a case study for those wishing to track donor funding for other specific population groups, diseases, or types of activities, which is of increasing interest given the growing focus on accountability and the pressure for detailed linking of funding with changes in intervention coverage or even impact. We have demonstrated that a key term search can be a relatively efficient approach to identify the number and value of records in the CRS database that include specific terms, but that doing so requires a thorough approach to maximize sensitivity and specificity and to reduce bias. Despite including additional donors, IHME's estimates of aid to MNCH are much lower than those of the Countdown in part because of their exclusion of health systems and sectoral funding, malaria, HIV, and non-communicable diseases from their definition of funding for MNCH, but also because their key terms are insensitive [17]. By reviewing our findings in detail, we were able to refine our search and also to separate those projects that exclusively targeted newborns from those that mentioned newborns alongside other population groups. Our approach of combining key terms with a review of individual records was nonetheless far less time-consuming than the approach employed by the Countdown to 2015, which entailed individually reviewing and coding many hundreds of thousands of records. The methods presented here may therefore provide an effective complementary approach to track trends in funding to specific areas, while continuing to use the Countdown framework to provide a more accurate estimate of the total value of aid to MNCH.

In addition to those already described, our key term approach has several further limitations. While we worked hard to develop a highly sensitive list of key terms, records containing typographical errors may have been missed and some useful key terms may have been omitted. Our analysis was also constrained by the CRS data to which it was applied [19]. This database does not capture all aid flows, as emerging donors such as China, Brazil, India, and many wealthy Arab states, as well as private foundations, do not report their aid, although the Gates Foundation, the United Arab Emirates, and Kuwait are recent welcome exceptions. Our analysis is based only on data until 2010, as data on aid disbursements at the project or even sectoral level are not available until 12 mo following year end (and are often substantially revised in the following quarter). Furthermore, a number of donors who do report, including the Gates Foundation and the GAVI Alliance, have not retrospectively updated their reporting to 2002, which poses obstacles to analyses of aid trends. As this analysis was conceived as an analysis of aid to developing countries rather than of research funding, it relied on the CRS aid activities database, to which most of the major potential funders of research for newborns in developing countries, be they private foundations such as the Wellcome Trust, or even government research funding bodies such as the National Institutes of Health (US), the European Union's Directorate-General for Research and Innovation, and the Medical Research Council (UK), do not report. While some research funders provide public data on their funding, many do not, and for those that do, a wide range of issues would need to be addressed for this information to be analysed, including the lack of direct comparability of the data, whether to include and how to define pre-clinical and operational research, and, most importantly, how to define and identify research relevant to developing countries.

In response to increasing calls for analyses of funding to specific diseases and population groups [46], care must be taken to avoid over-interpretation of findings and to acknowledge the interdependence of health sector activities [47]. Analyses should be based on conceptually coherent frameworks, which recognise that categorisation of activities according to health system functions [48], ingredients, and level, as well as disease and population group, each necessarily overlap. While comparisons of categories within such concepts may be informative (e.g., stewardship versus service delivery, medicines versus health workers, primary versus tertiary level care, malaria versus HIV, or newborns versus adult men), comparisons of funding across conceptual boundaries (e.g., health workers versus HIV, or newborns versus malaria) are illogical and provide little clear insight. Newborn survival relies on a well-functioning health system with effective community, primary, and secondary-level care, and newborn health is affected by a number of communicable and non-communicable diseases. While our estimates do not take into account the value of aid supporting the wider health system, there is little evidence that donors are addressing newborns' needs given their share of the mortality burden relative to other population groups and the existence of cost-effective interventions, which is both inequitable and inefficient.

Going forwards, any form of key term search will be both limited by donors' descriptions and also highly vulnerable to gaming or active manipulation by donors. If donors are aware that they will be monitored for their inclusion of particular key terms in their funding descriptions and that no safeguards are in place to ensure that these key terms accurately reflect the substance of the activities they fund, they will face incentives to increase their use of such key terms without necessarily improving the detail and accuracy of their reporting or changing the substance of the activities they fund. We therefore recommend that the Development Assistance Committee take forward existing recommendations to allow records to reflect their multiple purposes [22], potentially according to the five categories we outline above.

### Conclusion

We find that our key term search of the CRS database was a time-efficient, sensitive, and specific method for estimating the value of aid disbursements explicitly mentioning newborn health activities, and that individually reviewing the records allowed us to improve the specificity of our search terms and to identify the value of projects exclusively benefitting newborns. We conclude that a critically developed and refined key term approach can be an effective complementary method to track trends in funding explicitly targeting specific areas, but that the Countdown framework, which takes into account health systems funding, provides a more accurate estimate of the total value of aid of benefit to mothers, newborns, and children as a whole. We recommend that groups seeking to track funding for other specific population groups, diseases, or types of activities consider a key term approach along with a detailed review of their findings, but also that they develop both their search terms and interpretations carefully, taking into account the limitations of funding descriptions, the degree of integration of their area of interest within others, the role of health systems funding, and the risk of future gaming inherent in any key term approach.

We provide evidence of a substantial rise in the value and proportion of aid disbursements mentioning newborns and newborn interventions from 2002 to 2010 and especially since around 2005, which we interpret as strong evidence that donor attention to newborn survival has increased. While the total value of aid actually benefitting newborns could conceivably be as high as a large proportion of the value of aid for MNCH, which the Countdown estimated at US$5.3 billion for 2008 (constant 2010 US$), it could also be as low as the US$5.7 million for non-research projects exclusively benefitting newborns. The degree to which newborn health and activities are necessarily integrated with maternal health and activities and, to a lesser extent, activities for post-neonatal children, causes wide uncertainty in the estimates and may vary from country to country [3]. Nonetheless, it appears unlikely that donor investment in newborn survival is commensurate with the 3.0 million newborn deaths worldwide each year and newborns' 43% share of under-five child mortality [5], and we provide strong evidence that donors have neglected to address the estimated 2.1 to 3.8 million stillbirths each year [10]. The leadership shown by the small handful of donors providing funding mentioning newborns is laudable, but an expansion of programmatic funding from a wider range of donors is urgently required to catalyze scale-up of cost-effective interventions to save newborn lives and meet MDG4 in 3 y. Ultimately, however, the responsibility lies with ministries of health to promote and protect the health of their populations, especially the most vulnerable. Newborns require vigilant monitoring by governments, donors, researchers, and non-governmental organizations to ensure that they receive due attention in the implementation of integrated MNCH projects and programmes, to ensure that their increasing mentions in aid records reflects substantive actions to improve their health.

## Supporting Information

Alternative Language Abstract S1 French translation of the abstract by CP.
(PDF)

Text S1 Supplementary information on methods and additional results.
(PDF)

## Abbreviations

CRS: Creditor Reporting System
IHME: Institute for Health Metrics and Evaluation
MDG: Millennium Development Goal
MNCH: maternal, newborn, and child health
MNH: maternal and newborn health
ODA: official development assistance
UNICEF: United Nations Children's Fund
