# Supplementary material for: Donor Funding for Newborn Survival: An Analysis of Donor-Reported Data, 2002–2010
Source: PLoS Med. 2012 Oct 30;9(10):e1001332. doi: 10.1371/journal.pmed.1001332 (PMC3484125; doi:10.1371/journal.pmed.1001332)
Supplement: Alternative Language Abstract S1 — French translation of the abstract by CP. (PDF) [file pmed.1001332.s001.pdf]

Le financement des bailleurs de fonds pour la survie du nouveau-né, 2002 à 2010 : Développement et application d'une méthode pour comptabiliser l'aide à un groupe cible spécifique dans les données notifiées par les donateurs

Catherine Pitt[1], Joy E Lawn[2], Meghna Ranganathan[1], Anne Mills[1], Kara Hanson[1]

[1] Department of Global Health & Development, London School of Hygiene & Tropical Medicine, London, United Kingdom
[2] Saving Newborn Lives/Save the Children, Cape Town, South Africa

Contexte
La mortalité néonatale représente 43% des morts des moins de cinq ans globalement et diminue plus lentement que la mortalité maternelle ou de l'enfant. Le financement des donateurs a augmenté en faveur de la santé maternelle, néonatale, et de l'enfant (SMNE), mais aucune analyse n'a distingué l'aide au nouveau-né. Nous avons évalué si et comment on pouvait tracer les flux de l'aide en faveur du nouveau-né, examiné les changements pendant la dernière décennie, et réfléchi sur les implications méthodologiques pour tracer le financement en faveur des populations ou maladies spécifiques.

Méthodes et Résultats
Nous avons revu et catégorisé des analyses précédentes de l'aide en faveur des populations, maladies, ou types d'activités spécifiques. On a ensuite développé et raffiné des termes clés associés à la survie du nouveau-né dans sept langues et recherché ces termes dans les titres et descriptions des versements des donateurs dans la base de données sur les activités d'aide du Système de Notification des Pays Créanciers (SNPC) de l'Organisation de Coopération et de Développement Economiques pour les années 2002 à 2010. Nous avons comparé les résultats avec la base de données du Compte à rebours 2015 de l'aide pour la SMNE (2003-2008) et la stratégie de recherche employée par l'Institute for Health Metrics and Evaluation. Avant 2005, les termes clés associés au nouveau-né étaient rares dans les versements, mais leur fréquence a sensiblement augmenté par la suite. On a trouvé seulement deux mentions du « mort-né » et seulement neuf références au « fœtus » dans toute variante d'orthographe et toute langue. La valeur totale des versements non-recherche qui mentionne tout terme de recherche du nouveau-né a crû de $38,4 millions en 2002 à $717,1m en 2010 (USD réel 2010). La valeur des projets non-recherche à profit exclusif du nouveau-né a fluctué un peu, mais est restée basse, à $5,7m en 2010. Les Etats-Unis et l'Unicef ont fourni la plus grande valeur de financement qui mentionne et qui bénéficie au nouveau-né, respectivement.

Conclusions
L'attention des bailleurs de fonds à la survie du nouveau-né a augmenté, mais il semble peu probable que l'aide soit à la mesure des 3,0 million de morts néonatales et 2,7 million de mort-nés chaque année. Nous recommandons que ceux qui comptabilisent et suivent l'aide aux autres groupes, maladies, ou activités spécifiques considèrent l'emploi des termes clés de recherche dans le SNPC à côté d'une revue détaillée de leurs données, mais aussi qu'ils développent leurs termes de recherche et leurs interprétations soigneusement, prenant en compte les limitations ci-décrites.
