# Supplementary material for: Donor Funding for Newborn Survival: An Analysis of Donor-Reported Data, 2002–2010
Source: PLoS Med. 2012 Oct 30;9(10):e1001332. doi: 10.1371/journal.pmed.1001332 (PMC3484125; doi:10.1371/journal.pmed.1001332)
Supplement: Text S1 — Supplementary information on methods and additional results. (PDF) [file pmed.1001332.s002.pdf]

Tracking aid for a specific target group: Donor funding for newborn survival, 2002-2010

SUPPLEMENTARY MATERIAL

Catherine Pitt[1]*, Joy E Lawn[2], Meghna Ranganathan[1], Anne Mills[1], Kara Hanson[1]

[1] Department of Global Health & Development, London School of Hygiene & Tropical Medicine, London, United Kingdom
[2] Saving Newborn Lives/Save the Children, Cape Town, South Africa

*Corresponding author: catherine.pitt@lshtm.ac.uk

Contents

Part I: Supplementary information on methods....3

Additional information on population estimates and deflators 3

Table 1. Definitions of aid exclusively benefitting newborns, also benefitting other population groups, and misclassified records....4

Table 2. Definitions of aid supporting research or non-research activities 4

Principles for coding....4

Table 3. Creditor Reporting System Sector Codes....5

Table 4. Details of Creditor Reporting System Sector and Purpose Codes Most Relevant to Health....6

Table 5. IHME Search Terms for Maternal, Newborn, and Child Health....9

Part II: Supplementary results 10

Table 6. Aid mentioning and exclusively benefitting newborns by percent in the most relevant sector codes..... 10

Table 7. Aid mentioning and exclusively benefitting newborns for research or non-research activities by whether donors report in all years..... 11

Table 8. Aid mentioning and exclusively benefitting newborns by year and donor 12

Table 9. Aid supporting research and non-research activities by year and donor.... 13

Table 10. Search terms not found in any records (English, German, French) 14

Table 11. Search terms not found in any records (Portuguese, Spanish, Italian, Dutch).... 15

Table 12. Titles and descriptions of misclassified records..... 16

Table 13. Titles and descriptions of non-research projects exclusively benefitting newborns. .. 20

Table 14. Titles and descriptions of research activities mentioning newborns but also benefiting other population groups..... 25

Table 15. Titles and descriptions of research activities exclusively benefitting newborns. 33

Table 16. Inter-rater reliability. 37

Part III: References.... 38

Part I: Supplementary information on methods

Additional information on population estimates and deflators

Population estimates

Estimates for the number of livebirths for the calculations of aid mentioning and exclusively benefitting newborns per live birth are based on analyses of data in World Population Prospects: the 2008 revision population database.[1] Data were downloaded for the medium fertility assumptions. The number of births in each recipient country each year were estimated as the product of the crude fertility rate for the relevant 5-year time period (2000-05 or 2005-10) and the total population in that country that year. As World Population Prospects only provides the estimated total population at 5-year intervals (i.e. for 2000, 2005, and 2010), annual populations estimates were generated by combining these 5-yearly population estimates with the growth rates for each of the two five-year periods using the standard exponential growth formula. Thus, for example:

$$Pop_b = Pop_a * e^{(b-a)*GrowthRate}$$

Where,
Pop_b = our estimate of the total population in a given year b
Pop_a = the estimated total population provided for 2000 or 2005, whichever is later while still preceding the year b
GrowthRate = the estimated total population growth rate provided for the 5-year period including year b.

This formula was also used to cross-check the population estimates provided at 5-yearly intervals and the population growth rates provided and found to be consistent.

Deflators

All data are presented in constant 2010 USD. The conversion between currencies and from current to constant dollars was performed using the same methods and deflators as used by the OECD. [2] Separate, country-specific deflators were used for each of the DAC member countries, reflecting the opportunity cost to each donor country of their contribution. An aggregate "Total DAC" deflator was applied to all multilateral institutions and global health initiatives, as well as to non-DAC bilateral donors (Kuwait and the United Arab Emirates). As the Bill & Melinda Gates Foundation is based in the United States, the United States' deflators are used for its contributions.

As IHME estimates for the value of aid to MNCH were not disaggregated by donor, donor-specific deflators could not be used and so the Total DAC deflator was used instead to convert from 2008 constant dollars (which IHME reported) to constant 2010 dollars as presented in figure 2 in the main paper.

Table 1. Definitions of aid exclusively benefitting newborns, also benefitting other population groups, and misclassified records

Coding category	Definition
**Exclusively benefitting newborns**	Activities whose sole aim is to maintain or improve the health of newborns or of the developing fetus.
**Mentions newborns but also benefits other population groups**	Activities which may maintain or improve the health of newborns or of the developing fetus, but which are also expected to improve the health of other population groups, such as mothers or post-neonatal children. These include, for example, activities to combat malaria in pregnancy, which benefits both the mother and developing fetus. They also include activities that take place in the neonatal period, such as BCG immunization, but whose benefits are felt throughout childhood and beyond. Support of breastfeeding in general is also included, as it is assumed that the activities include support for postneonatal children unless the project explicitly targets interventions to support early breastfeeding for the benefit of newborns.
**Misclassified**	Activities which could clearly in no way benefit newborns. These include, for example, records identified by search terms because newborns are mentioned as part of the situation analysis but will not benefit from the activities, where acronyms are used with a different meaning intended, or research projects using fetal stem cells which are not aiming to promote perinatal health.

Table 2. Definitions of aid supporting research or non-research activities

Coding category	Definition
**Research**	Activities whose primary purpose is to generate new knowledge or to support research infrastructure. While a portion of these activities may include support for the implementation of an intervention being tested, the funding primarily supports evaluation activities. These include, for example, efficacy and effectiveness trials, epidemiological studies, studies of mechanisms of disease and biological pathways, and publications in academic journals. In addition, institutional support to research institutes are also included.
**Not research**	All other activities, including support to the implementation of projects and programmes, policy change, and advocacy activities.

Principles for coding
The following principles are based on those previously used for the Countdown to 2015 analysis.[3]

- Coding is primarily based on four key fields: purpose code, project title, short description, and long description, but can also include data on the donor and recipient.
- Assume that the long description is correct if it specifies but does not contradict the other 3 fields (e.g. specifies “promotion of early and exclusive breastfeeding for newborns delivered in health facilities” on a project otherwise described as “breastfeeding”)
- Where one of the four key fields directly contradict the others, assume that it is a mistake and base coding on the remaining fields.

Table 3. Creditor Reporting System Sector Codes
This table lists all the sector codes in the CRS database [4] and for each provides a complete list of the purpose codes falling within that sector code. All sectors were included in the final search for newborn key terms except for action relating to debt (i.e. debt forgiveness)
.

Sector code	Name of sector code	Purpose codes within this sector code	Newborn Search
**111**	Education, level unspecified	11110, 11120, 11130, 11182	Yes
**112**	Basic education	11220, 11230, 11240	Yes
**113**	Secondary education	11320, 11330	Yes
**114**	Post-secondary education	11420, 11430	Yes
**121**	Health, general	12110, 12181, 12182, 12191	Yes
**122**	Basic health	12220, 12230, 12240, 12250, 12261, 12262, 12263, 12281	Yes
**130**	POPULATION POLICIES/PROGRAMMES AND REPRODUCTIVE HEALTH	13010, 13020, 13030, 13040, 13081	Yes
**140**	WATER AND SANITATION	14010, 14015, 14020, 14021, 14022, 14030, 14031, 14032, 14040, 14050, 14081	Yes
**151**	Government and civil society, general	15110, 15111, 15112, 15113, 15130, 15150, 15151, 15152, 15153, 15160, 15170	Yes
**152**	Conflict prevention and resolution, peace and security	15210, 15220, 15230, 15240, 15250, 15261	Yes
**160**	OTHER SOCIAL INFRASTRUCTURE AND SERVICES	16010, 16020, 16030, 16040, 16050, 16061, 16062, 16063, 16064	Yes
**210**	TRANSPORT AND STORAGE	21010, 21020, 21030, 21040, 21050, 21061, 21081	Yes
**220**	COMMUNICATION	22010, 22020, 22030, 22040	Yes
**230**	ENERGY GENERATION AND SUPPLY	23010, 23020, 23030, 23040, 23050, 23061, 23062, 23063, 23064, 23065, 23066, 23067, 23068, 23069, 23070, 23081, 23082	Yes
**240**	BANKING AND FINANCIAL SERVICES	24010, 24020, 24030, 24040, 24081	Yes
**250**	BUSINESS AND OTHER SERVICES	25010, 25020	Yes
**311**	AGRICULTURE	31110, 31120, 31130, 31140, 31150, 31161, 31162, 31163, 31164, 31165, 31166, 31181, 31182, 31191, 31192, 31193, 31194, 31195	Yes
**312**	FORESTRY	31210, 31220, 31261, 31281, 31291	Yes
**313**	FISHING	31310, 31320, 31381, 31382	Yes
**321**	INDUSTRY	32110, 32120, 32130, 32140, 32162, 32163, 32164, 32165, 32166, 32167, 32168, 32169, 32170, 32171, 32172, 32182	Yes
**322**	MINERAL RESOURCES AND MINING	32210, 32220, 32261, 32262, 32264, 32265, 32266, 32267, 32268	Yes
**323**	CONSTRUCTION	32310	Yes
**331**	TRADE POLICY AND REGULATIONS AND TRADE-RELATED ADJUSTMENT	33110, 33120, 33130, 33140, 33181	Yes
**332**	TOURISM	33210	Yes
**410**	General environmental protection	41010, 41020, 41030, 41040, 41081, 41082	Yes
**430**	Other multisector	43010, 43030, 43040, 43050, 43082	Yes
**510**	General budget support	51010	Yes
**520**	Developmental food aid/Food security assistance	52010	Yes
**530**	Other commodity assistance	53030, 53040	Yes
**600**	ACTION RELATING TO DEBT	60010, 60020, 60030, 60040, 60062, 60063	No
**720**	Emergency Response	72010, 72040, 72050	Yes
**730**	Reconstruction relief and rehabilitation	73010	Yes
**740**	Disaster prevention and preparedness	74010	Yes
**910**	ADMINISTRATIVE COSTS OF DONORS	91010	Yes
**930**	REFUGEES IN DONOR COUNTRIES	93010	Yes
**998**	UNALLOCATED/ UNSPECIFIED	99810, 99820	Yes

Table 4. Details of Creditor Reporting System Sector and Purpose Codes Most Relevant to Health
The following table reproduces exactly the sections of the OECD's list of sector and purpose codes most relevant to health. Please not that, as explained in *Webtable 2*, additional sectors were also included in the search strategy to identify records aiming to benefit newborn health. Please see the OECD website for further information on purpose codes: http://www.oecd.org/document/21/0,3746,en_2649_34447_1914325_1_1_1_1,00.html

DAC 5 / SECTOR CODE	CRS PURPOSE CODE	DESCRIPTION	Clarifications / Additional notes on coverage
**120**		**HEALTH**	
**121**		**Health, general**	
	**12110**	Health policy and administrative management	Health sector policy, planning and programmes; aid to health ministries, public health administration; institution capacity building and advice; medical insurance programmes; unspecified health activities.
	**12181**	Medical education/training	Medical education and training for tertiary level services.
	**12182**	Medical research	General medical research (excluding basic health research).
	**12191**	Medical services	Laboratories, specialised clinics and hospitals (including equipment and supplies); ambulances; dental services; mental health care; medical rehabilitation; control of non-infectious diseases; drug and substance abuse control [excluding narcotics traffic control (16063)].
**122**		**Basic health**	
	**12220**	Basic health care	Basic and primary health care programmes; paramedical and nursing care programmes; supply of drugs, medicines and vaccines related to basic health care.
	**12230**	Basic health infrastructure	District-level hospitals, clinics and dispensaries and related medical equipment; excluding specialised hospitals and clinics (12191).
	**12240**	Basic nutrition	Direct feeding programmes (maternal feeding, breastfeeding and weaning foods, child feeding, school feeding); determination of micro-nutrient deficiencies; provision of vitamin A, iodine, iron etc.; monitoring of nutritional status; nutrition and food hygiene education; household food security.
	**12250**	Infectious disease control	Immunisation; prevention and control of infectious and parasite diseases, except malaria (12262), tuberculosis (12263), HIV/AIDS and other STDs (13040). It includes diarrheal diseases, vector-borne diseases (e.g. river blindness and guinea worm), viral diseases, mycosis, helminthiasis, zoonosis, diseases by other bacteria and viruses, pediculosis, etc.
	**12261**	Health education	Information, education and training of the population for improving health knowledge and practices; public health and awareness campaigns; promotion of improved personal hygiene practices, including use of sanitation facilities and handwashing with soap.
	**12262**	Malaria control	Prevention and control of malaria.
	**12263**	Tuberculosis control	Immunisation, prevention and control of tuberculosis.
	**12281**	Health personnel development	Training of health staff for basic health care services.

SECTOR CODE	CRS PURPOSE CODE	DESCRIPTION	Clarifications / Additional notes on coverage
**130**		**POPULATION POLICIES/PROGRAMMES AND REPRODUCTIVE HEALTH**	
	**13010**	Population policy and administrative management	Population/development policies; census work, vital registration; migration data; demographic research/analysis; reproductive health research; unspecified population activities.
	**13020**	Reproductive health care	Promotion of reproductive health; prenatal and postnatal care including delivery; prevention and treatment of infertility; prevention and management of consequences of abortion; safe motherhood activities.
	**13030**	Family planning	Family planning services including counselling; information, education and communication (IEC) activities; delivery of contraceptives; capacity building and training.
	**13040**	STD control including HIV/AIDS	All activities related to sexually transmitted diseases and HIV/AIDS control e.g. information, education and communication; testing; prevention; treatment, care.
	**13081**	Personnel development for population and reproductive health	Education and training of health staff for population and reproductive health care services.

**700**		**HUMANITARIAN AID**	Within the overall definition of ODA, humanitarian aid is assistance designed to save lives, alleviate suffering and maintain and protect human dignity during and in the aftermath of emergencies. To be classified as humanitarian, aid should be consistent with the humanitarian principles of humanity, impartiality, neutrality and independence.
**720**		**Emergency Response**	An emergency is a situation which results from man made crises and/or natural disasters.
	**72010**	Material relief assistance and services	Shelter, water, sanitation and health services, supply of medicines and other non-food relief items; assistance to refugees and internally displaced people in developing countries other than for food (72040) or protection (72050).
	**72040**	Emergency food aid	Food aid normally for general free distribution or special supplementary feeding programmes; short-term relief to targeted population groups affected by emergency situations. Excludes non-emergency food security assistance programmes/food aid (52010).
	**72050**	Relief co-ordination; protection and support services	Measures to co-ordinate delivery of humanitarian aid, including logistics and communications systems; measures to promote and protect the safety, well-being, dignity and integrity of civilians and those no longer taking part in hostilities. (Activities designed to protect the security of persons or property through the use or display of force are not reportable as ODA.)
**730**		**Reconstruction relief and rehabilitation**	This relates to activities during and in the aftermath of an emergency situation. Longer-term activities to improve the level of infrastructure or social services should be reported under the relevant economic and social sector codes. See also guideline on distinguishing humanitarian from sector-allocable aid.
	**73010**	Reconstruction relief and rehabilitation	Short-term reconstruction work after emergency or conflict limited to restoring pre-existing infrastructure (e.g. repair or construction of roads, bridges and ports, restoration of essential facilities, such as water and sanitation, shelter, health care services); social and economic rehabilitation in the aftermath of emergencies to facilitate transition and enable populations to return to their previous livelihood or develop a new livelihood in the wake of an emergency situation (e.g. trauma counselling and treatment, employment programmes).
**740**		**Disaster prevention and preparedness**	See codes 41050 and 15220 for prevention of floods and conflicts.
	**74010**	Disaster prevention and preparedness	Disaster risk reduction activities (e.g. developing knowledge, natural risks cartography, legal norms for construction); early warning systems; emergency contingency stocks and contingency planning including preparations for forced displacement.

Table 5. IHME Search Terms for Maternal, Newborn, and Child Health
The following list of search terms and accompanying text are reproduced from the methods annex to a report by the Institute for Health Metrics and Evaluation [5] on which certain findings in a comment in the *Lancet* are based [6]. All of these search terms were applied to the titles and descriptions of records identified by our newborn search terms, after removing accents from the latter. We calculate the sensitivity of these search terms to identify ODA records targeting newborns compared with our own search strategy by calculating the % (by number and value of records) of records identified by our search strategy that are also identified by at least one of the search terms listed below.

Source: Table 8.1 (p.39), Institute for Health Metrics and Evaluation (2010). Financing global health 2010: Development assistance and country spending in economic uncertainty: Methods Annex. Seattle, WA, IHME. Available at: http://www.healthmetricsandevaluation.org/sites/default/files/policy_report/2010/financing_global_health_2010_methods_IHME.pdf

Reproduced from source:
"To identify health aid for HIV/AIDS; tuberculosis; malaria; health sector support; maternal, newborn, and child health; and noncommunicable diseases, we searched for keywords associated with each in descriptive fields of our IHME DAH Database (Country and Regional Recipient Level), as shown in Table 8.1. This includes a subset of global health channels for which information on country and/or regional allocation was available, namely the bilateral development assistance agencies from the 23 DAC member countries, the EC, GFATM, GAVI, the World Bank, ADB, IDB, AfDB, and BMGF. When a project was matched to two or more areas, the dollar value of the grant was divided evenly across the matched areas."

"Note: When conducting the keyword search, we capitalized all project descriptions and search terms, which eliminated all accents from the text. Thus, our French search terms are listed without accents."

In the 2011 version of the report [7], from which the IHME estimates of aid to MNCH presented in **figures 2a** and **2b** are taken, the following statement was added:
"*For projects that matched to both MNCH and either HIV or malaria, MNCH was removed as a concurrent health focus, based on the observation that many project descriptions for HIV and malaria programs incorrectly appear MNCH-related."*

accoucheur qualifie	personnel de sante qualifie
antenatal care	planification familiale
Antenatal	planning familial
child health	postpartum
child mortality	prenatal
child survival	reproductive health
deces maternel	safe motherhood
emergency obstetric care	sante de l'enfant
family planning	sante de la mere, du nouveau-ne et de l'enfant
infant mortality	sante du nouveau-ne
integrated management of childhood illness	sante genesique
maternal and infant health	sante maternelle
maternal death	sante reproductive
maternal health	sba
maternal mortality	skilled birth attendant
maternal, newborn & child health	soins obstetriques d'urgence
mortalite des enfants	soins obstetriques essentiels
mortalite des moins de cinq ans	soins prenatals
mortalite infantile	under-five mortality
mortalite maternelle	vaccination
neonatal	vitamin a
newborn health	vitamine a
perinatal	

Part II: Supplementary results

Table 6. Aid mentioning and exclusively benefitting newborns by percent in the most relevant sector codes.

	2002		2003		2004		2005		2006		2007		2008		2009		2010		TOTAL, 2002-10	
	Mentions newborns	Exclusively benefits newborns	Mentions newborns	Exclusively benefits newborns	Mentions newborns	Exclusively benefits newborns	Mentions newborns	Exclusively benefits newborns	Mentions newborns	Exclusively benefits newborns	Mentions newborns	Exclusively benefits newborns	Mentions newborns	Exclusively benefits newborns	Mentions newborns	Exclusively benefits newborns	Mentions newborns	Exclusively benefits newborns	Mentions newborns	Exclusively benefits newborns
**By number of records**																				
**Health (code 120)**	62.4%	40.0%	66.7%	71.4%	74.1%	60.0%	66.7%	60.0%	60.5%	6.5%	46.1%	5.4%	27.9%	6.1%	22.5%	9.2%	17.1%	12.0%	29.2%	10.5%
**Population policies and programmes and reproductive health (code 130)**	28.0%	50.0%	21.4%	28.6%	10.3%	40.0%	19.5%	40.0%	33.3%	88.3%	50.5%	91.3%	68.5%	93.9%	75.9%	90.8%	80.8%	86.7%	67.5%	87.5%
**Emergency response (code 720)**	1.1%	10.0%	1.2%	0.0%	1.7%	0.0%	0.0%	0.0%	0.7%	0.0%	0.7%	0.0%	0.7%	0.0%	0.9%	0.0%	0.7%	0.0%	0.8%	0.2%
**Other sectors**	8.6%	0.0%	10.7%	0.0%	13.8%	0.0%	13.8%	0.0%	5.4%	5.2%	2.8%	3.3%	2.9%	0.0%	0.7%	0.0%	1.4%	1.3%	2.6%	1.8%
**By value of records**																				
**Health (code 120)**	57.5%	79.9%	66.7%	49.0%	79.4%	75.2%	68.3%	54.0%	71.3%	47.5%	71.2%	37.8%	29.1%	30.4%	27.2%	71.1%	23.0%	10.6%	38.9%	50.7%
**Population policies and programmes and reproductive health (code 130)**	33.6%	18.9%	14.7%	51.0%	3.0%	24.8%	13.7%	46.0%	15.9%	38.3%	25.8%	51.2%	66.9%	69.6%	72.3%	28.9%	75.5%	89.4%	56.3%	47.6%
**Emergency response (code 720)**	0.2%	1.2%	1.2%	0.0%	0.2%	0.0%	0.0%	0.0%	0.7%	0.0%	0.8%	0.0%	0.3%	0.0%	0.4%	0.0%	1.1%	0.0%	0.7%	0.1%
**Other sectors**	8.7%	0.0%	17.5%	0.0%	17.4%	0.0%	18.0%	0.0%	12.1%	14.3%	2.1%	11.0%	3.7%	0.0%	0.0%	0.0%	0.5%	0.0%	4.1%	1.6%

Table 7. Aid mentioning and exclusively benefitting newborns for research or non-research activities by whether donors report in all years. This table provides the data used in figure 1 in the manuscript in constant 2010 USD millions.

			2002	2003	2004	2005	2006	2007	2008	2009	2010	Total
Donors reporting in at least one but not all years	Research	exclusively benefiting newborns	0.0	0.0	0.0	0.0	0.0	0.0	0.0	46.8	19.4	66.2
		mentioning newborns but also benefiting other populations	0.0	0.0	0.0	0.0	0.0	0.0	0.0	62.7	38.8	101.4
	Not research	exclusively benefiting newborns	0.3	0.1	0.0	0.0	0.2	0.6	0.1	0.0	1.6	2.9
		mentioning newborns but also benefiting other populations	0.0	0.0	0.0	0.0	0.0	52.8	12.2	37.1	33.8	135.9
Donors reporting in all years	Research	exclusively benefiting newborns	0.1	0.0	0.0	0.2	0.0	1.1	0.3	0.0	0.0	1.6
		mentioning newborns but also benefiting other populations	0.0	0.0	0.0	0.3	1.1	0.9	0.2	1.4	2.0	5.9
	Not research	exclusively benefiting newborns	4.8	2.3	3.0	2.4	6.4	6.8	7.5	5.5	4.2	42.9
		mentioning newborns but also benefiting other populations	33.3	70.2	72.4	153.2	212.7	126.7	348.7	499.7	677.6	2,194.5

Table 8. Aid mentioning and exclusively benefitting newborns by year and donor
Aid that "exclusively benefits newborns" is a subset of Aid that "mentions newborns". Constant 2010 USD millions.

	2002		2003		2004		2005		2006		2007		2008		2009		2010		TOTAL	
	Mentions newborns	Exclusively benefits newborns	Mentions newborns	Exclusively benefits newborns	Mentions newborns	Exclusively benefits newborns	Mentions newborns	Exclusively benefits newborns	Mentions newborns	Exclusively benefits newborns	Mentions newborns	Exclusively benefits newborns	Mentions newborns	Exclusively benefits newborns	Mentions newborns	Exclusively benefits newborns	Mentions newborns	Exclusively benefits newborns	Mentions newborns	Exclusively benefits newborns
**BILATERAL DONORS**	**16.8**	**5.1**	**37.8**	**2.4**	**27.8**	**3.0**	**40.3**	**2.6**	**42.1**	**4.9**	**82.8**	**5.4**	**297.4**	**5.5**	**488.6**	**3.6**	**619.4**	**0.8**	**1653.0**	**33.4**
Australia	0.3	0.0	0.3	0.0	0.4	0.3	0.5	0.3	1.5	0.3	3.2	0.2	11.6	0.0	29.2	0.0	37.0	0.0	83.9	1.0
Austria	0.0	0.0	0.0	0.0	0.0	0.0	0.1	0.0	0.0	0.0	0.0	0.0	0.0	0.0	0.0	0.0	0.0	0.0	0.1	0.0
Belgium	1.3	0.0	1.3	0.0	0.0	0.0	0.3	0.0	1.3	0.9	2.7	0.9	0.6	0.0	0.0	0.0	0.1	0.1	7.5	1.9
Canada	3.0	0.0	25.3	0.0	23.0	0.0	21.6	0.0	21.9	0.0	27.0	0.0	28.4	0.0	22.1	0.0	36.2	0.0	208.6	0.0
Denmark	NR	NR	0.0	0.0	0.0	0.0	0.0	0.0	0.0	0.0	0.0	0.0	0.1	0.0	0.0	0.0	0.0	0.0	0.1	0.0
Finland	0.3	0.3	0.1	0.1	NR	NR	NR	NR	0.2	0.2	0.2	0.2	0.1	0.1	0.1	0.0	0.0	0.0	0.9	0.8
France	0.0	0.0	0.0	0.0	0.0	0.0	0.0	0.0	0.0	0.0	0.0	0.0	0.2	0.0	1.9	0.0	0.2	0.0	2.3	0.1
Germany	5.4	0.0	5.8	0.0	0.2	0.0	0.0	0.0	0.0	0.0	0.0	0.0	0.0	0.0	0.1	0.0	1.9	0.0	13.4	0.0
Greece	0.0	0.0	0.0	0.0	0.2	0.2	0.0	0.0	0.0	0.0	0.0	0.0	0.0	0.0	0.0	0.0	0.0	0.0	0.2	0.2
Ireland	0.0	0.0	0.0	0.0	0.0	0.0	0.0	0.0	0.0	0.0	0.2	0.0	0.2	0.0	0.0	0.0	0.0	0.0	0.3	0.0
Italy	0.3	0.0	0.0	0.0	0.0	0.0	0.0	0.0	0.0	0.0	0.4	0.0	0.3	0.2	0.7	0.0	0.3	0.2	2.0	0.5
Japan	3.8	3.8	0.7	0.7	2.0	2.0	0.0	0.0	0.0	0.0	0.0	0.0	1.3	0.0	0.8	0.0	3.1	0.0	11.6	6.6
Korea	NR	NR	NR	NR	NR	NR	NR	NR	0.0	0.0	0.5	0.5	1.1	0.0	0.0	0.0	0.5	0.0	2.1	0.5
Luxembourg	0.0	0.0	0.0	0.0	0.0	0.0	0.0	0.0	0.0	0.0	0.0	0.0	0.0	0.0	0.0	0.0	2.8	0.0	2.8	0.0
Netherlands	0.8	0.0	2.3	0.0	1.1	0.0	0.0	0.0	1.7	0.0	3.0	0.0	5.6	0.0	5.6	0.0	4.5	0.0	24.6	0.0
New Zealand	0.0	0.0	0.0	0.0	0.0	0.0	0.0	0.0	0.0	0.0	0.0	0.0	0.0	0.0	1.3	0.0	0.5	0.0	1.7	0.0
Norway	0.0	0.0	0.0	0.0	0.0	0.0	0.0	0.0	0.0	0.0	0.8	0.0	4.9	0.3	12.6	0.0	20.7	0.2	39.2	0.5
Portugal	0.0	0.0	0.0	0.0	0.0	0.0	0.0	0.0	0.0	0.0	0.0	0.0	0.0	0.0	0.0	0.0	0.2	0.0	0.2	0.0
Spain	0.2	0.1	0.6	0.4	0.0	0.0	1.1	0.0	3.8	0.1	2.9	0.4	12.6	0.6	9.6	0.2	10.6	0.4	41.5	2.2
Sweden	0.4	0.0	0.1	0.0	0.0	0.0	0.0	0.0	0.3	0.0	0.1	0.0	0.7	0.0	1.5	0.0	2.3	0.0	5.4	0.0
Switzerland	0.0	0.0	0.0	0.0	0.0	0.0	1.2	1.2	2.8	2.8	2.0	2.0	1.6	1.6	0.8	0.8	5.7	0.0	14.0	8.3
United Kingdom	1.1	0.9	1.3	1.2	0.8	0.5	4.8	1.1	2.0	0.4	7.9	1.2	34.7	0.3	11.3	0.0	11.8	0.0	75.7	5.6
United States	0.0	0.0	0.0	0.0	0.0	0.0	10.7	0.0	6.6	0.3	32.0	0.0	193.4	2.4	391.1	2.5	481.1	0.0	1114.8	5.2
**MULTILATERAL DONORS**	**21.6**	**0.0**	**34.8**	**0.0**	**46.2**	**0.0**	**94.6**	**0.0**	**158.5**	**1.6**	**34.8**	**3.1**	**38.8**	**2.4**	**19.7**	**1.9**	**52.2**	**3.4**	**501.2**	**12.4**
EU Institutions	0.0	0.0	0.0	0.0	0.0	0.0	0.0	0.0	0.0	0.0	0.7	0.0	4.8	0.0	6.0	0.0	6.0	0.0	17.5	0.0
OFID	NR	NR	NR	NR	NR	NR	NR	NR	NR	NR	NR	NR	NR	NR	0.0	0.0	0.1	0.0	0.1	0.0
UNFPA	0.0	0.0	0.6	0.0	0.0	0.0	0.0	0.0	0.0	0.0	1.1	0.0	3.0	0.0	0.0	0.0	0.0	0.0	4.7	0.0
UNICEF	3.8	0.0	8.5	0.0	5.5	0.0	6.8	0.0	4.6	1.6	6.3	3.1	9.5	2.4	7.1	1.9	6.8	3.4	58.9	12.4
WHO	NR	NR	NR	NR	NR	NR	NR	NR	NR	NR	NR	NR	NR	NR	6.6	0.0	0.0	0.0	6.6	0.0
World Bank -IDA	17.8	0.0	25.7	0.0	40.7	0.0	87.8	0.0	153.9	0.0	26.9	0.0	21.4	0.0	0.0	0.0	39.3	0.0	413.4	0.0
**GLOBAL HEALTH INITIATIVES**	**0.0**	**0.0**	**0.0**	**0.0**	**1.5**	**0.0**	**21.1**	**0.0**	**19.7**	**0.0**	**71.3**	**0.0**	**32.8**	**0.0**	**5.1**	**0.0**	**12.8**	**0.0**	**164.3**	**0.0**
GAVI	NR	NR	NR	NR	NR	NR	NR	NR	NR	NR	52.8	0.0	10.9	0.0	0.1	0.0	-0.1	0.0	63.7	0.0
Global Fund	0.0	0.0	0.0	0.0	1.5	0.0	21.1	0.0	19.7	0.0	18.5	0.0	21.9	0.0	5.0	0.0	12.9	0.0	100.6	0.0
**PRIVATE DONORS**	**NR**	**NR**	**NR**	**NR**	**NR**	**NR**	**NR**	**NR**	**NR**	**NR**	**NR**	**NR**	**NR**	**NR**	**139.9**	**46.8**	**93.0**	**21.0**	**232.9**	**67.8**
Bill & Melinda Gates Foundation	NR	NR	NR	NR	NR	NR	NR	NR	NR	NR	NR	NR	NR	NR	139.9	46.8	93.0	21.0	232.9	67.8
**GRAND TOTAL**	**38.4**	**5.1**	**72.7**	**2.4**	**75.4**	**3.0**	**156.0**	**2.6**	**220.4**	**6.6**	**188.9**	**8.5**	**369.0**	**7.9**	**653.3**	**52.4**	**777.3**	**25.1**	**2551.4**	**113.6**

Table 9. Aid supporting research and non-research activities by year and donor
Constant 2010 USD millions.

	2002		2003		2004		2005		2006		2007		2008		2009		2010		TOTAL	
	Not research	Research	Not research	Research	Not research	Research	Not research	Research	Not research	Research	Not research	Research	Not research	Research	Not research	Research	Not research	Research	Not research	Research
**BILATERAL DONORS**	**16.7**	**0.1**	**37.8**	**0.0**	**27.8**	0.0	**39.9**	**0.5**	**41.0**	**1.1**	**80.8**	**1.9**	**296.9**	**0.5**	**487.2**	**1.4**	**617.4**	**2.0**	**1645.5**	**7.5**
**Australia**	0.3	0.0	0.3	0.0	0.4	0.0	0.5	0.0	1.5	0.0	3.2	0.0	11.6	0.0	29.2	0.0	37.0	0.0	83.9	0.0
**Austria**	0.0	0.0	0.0	0.0	0.0	0.0	0.1	0.0	0.0	0.0	0.0	0.0	0.0	0.0	0.0	0.0	0.0	0.0	0.1	0.0
**Belgium**	1.3	0.0	1.3	0.0	0.0	0.0	0.0	0.3	0.9	0.4	2.1	0.6	0.4	0.2	0.0	0.0	0.1	0.0	6.1	1.4
**Canada**	3.0	0.0	25.3	0.0	23.0	0.0	21.6	0.0	21.9	0.0	27.0	0.0	28.4	0.0	22.1	0.0	36.2	0.0	208.6	0.0
**Denmark**	NR	NR	0.0	0.0	0.0	0.0	0.0	0.0	0.0	0.0	0.0	0.0	0.1	0.0	0.0	0.0	0.0	0.0	0.1	0.0
**Finland**	0.3	0.0	0.1	0.0	NR	NR	NR	NR	0.2	0.0	0.2	0.0	0.1	0.0	0.1	0.0	0.0	0.0	0.9	0.0
**France**	0.0	0.0	0.0	0.0	0.0	0.0	0.0	0.0	0.0	0.0	0.0	0.0	0.2	0.0	1.9	0.0	0.0	0.2	2.1	0.2
**Germany**	5.4	0.0	5.8	0.0	0.2	0.0	0.0	0.0	0.0	0.0	0.0	0.0	0.0	0.0	0.1	0.0	1.9	0.0	13.4	0.0
**Greece**	0.0	0.0	0.0	0.0	0.2	0.0	0.0	0.0	0.0	0.0	0.0	0.0	0.0	0.0	0.0	0.0	0.0	0.0	0.2	0.0
**Ireland**	0.0	0.0	0.0	0.0	0.0	0.0	0.0	0.0	0.0	0.0	0.0	0.2	0.2	0.0	0.0	0.0	0.0	0.0	0.2	0.2
**Italy**	0.3	0.0	0.0	0.0	0.0	0.0	0.0	0.0	0.0	0.0	0.4	0.0	0.3	0.0	0.7	0.0	0.3	0.0	2.0	0.0
**Japan**	3.8	0.0	0.7	0.0	2.0	0.0	0.0	0.0	0.0	0.0	0.0	0.0	1.3	0.0	0.8	0.0	3.1	0.0	11.6	0.0
**Korea**	NR	NR	NR	NR	NR	NR	NR	NR	0.0	0.0	0.5	0.0	1.1	0.0	0.0	0.0	0.5	0.0	2.1	0.0
**Luxembourg**	0.0	0.0	0.0	0.0	0.0	0.0	0.0	0.0	0.0	0.0	0.0	0.0	0.0	0.0	0.0	0.0	2.8	0.0	2.8	0.0
**Netherlands**	0.8	0.0	2.3	0.0	1.1	0.0	0.0	0.0	1.7	0.0	3.0	0.0	5.6	0.0	5.6	0.0	4.5	0.0	24.6	0.0
**New Zealand**	0.0	0.0	0.0	0.0	0.0	0.0	0.0	0.0	0.0	0.0	0.0	0.0	0.0	0.0	1.3	0.0	0.5	0.0	1.7	0.0
**Norway**	0.0	0.0	0.0	0.0	0.0	0.0	0.0	0.0	0.0	0.0	0.8	0.1	4.9	0.0	12.6	0.0	20.4	0.3	38.8	0.4
**Portugal**	0.0	0.0	0.0	0.0	0.0	0.0	0.0	0.0	0.0	0.0	0.0	0.0	0.0	0.0	0.0	0.0	0.2	0.0	0.2	0.0
**Spain**	0.2	0.0	0.6	0.0	0.0	0.0	1.1	0.0	3.8	0.1	2.9	0.0	12.6	0.0	9.6	0.0	10.6	0.0	41.4	0.2
**Sweden**	0.4	0.0	0.1	0.0	0.0	0.0	0.0	0.0	0.3	0.0	0.1	0.0	0.7	0.0	1.5	0.0	0.9	1.4	3.9	1.4
**Switzerland**	0.0	0.0	0.0	0.0	0.0	0.0	1.2	0.0	2.8	0.0	2.0	0.0	1.6	0.0	0.8	0.0	5.7	0.0	14.0	0.0
**United Kingdom**	1.0	0.1	1.3	0.0	0.8	0.0	4.6	0.2	2.0	0.0	6.8	1.1	34.4	0.3	9.9	1.4	11.8	0.0	72.6	3.0
**United States**	0.0	0.0	0.0	0.0	0.0	0.0	10.7	0.0	5.9	0.7	32.0	0.0	193.4	0.0	391.1	0.0	481.1	0.0	1114.2	0.7
**MULTILATERAL DONORS**	**21.6**	**0.0**	**34.8**	**0.0**	**46.2**	0.0	**94.6**	**0.0**	**158.5**	**0.0**	**34.8**	**0.0**	**38.8**	**0.0**	**19.3**	**0.4**	**52.2**	**0.0**	**500.8**	**0.4**
**EU Institutions**	0.0	0.0	0.0	0.0	0.0	0.0	0.0	0.0	0.0	0.0	0.7	0.0	4.8	0.0	6.0	0.0	6.0	0.0	17.5	0.0
**OFID**	NR	NR	NR	NR	NR	NR	NR	NR	NR	NR	NR	NR	NR	NR	0.0	0.0	0.1	0.0	0.1	0.0
**UNFPA**	0.0	0.0	0.6	0.0	0.0	0.0	0.0	0.0	0.0	0.0	1.1	0.0	3.0	0.0	0.0	0.0	0.0	0.0	4.7	0.0
**UNICEF**	3.8	0.0	8.5	0.0	5.5	0.0	6.8	0.0	4.6	0.0	6.3	0.0	9.5	0.0	7.1	0.0	6.8	0.0	58.9	0.0
**WHO**	NR	NR	NR	NR	NR	NR	NR	NR	NR	NR	NR	NR	NR	NR	6.2	0.4	0.0	0.0	6.2	0.4
**World Bank -IDA**	17.8	0.0	25.7	0.0	40.7	0.0	87.8	0.0	153.9	0.0	26.9	0.0	21.4	0.0	0.0	0.0	39.3	0.0	413.4	0.0
**GLOBAL HEALTH INITIATIVES**	**0.0**	**0.0**	**0.0**	**0.0**	**1.5**	0.0	**21.1**	**0.0**	**19.7**	**0.0**	**71.3**	**0.0**	**32.8**	**0.0**	**5.1**	**0.0**	**12.8**	**0.0**	**164.3**	**0.0**
**GAVI**	0.0	0.0	0.0	0.0	0.0	0.0	0.0	0.0	0.0	0.0	52.8	0.0	10.9	0.0	0.1	0.0	-0.1	0.0	63.7	0.0
**Global Fund**	0.0	0.0	0.0	0.0	1.5	0.0	21.1	0.0	19.7	0.0	18.5	0.0	21.9	0.0	5.0	0.0	12.9	0.0	100.6	0.0
**PRIVATE DONORS**	**NR**	**NR**	**NR**	**NR**	**NR**	**NR**	**NR**	**NR**	**NR**	**NR**	**NR**	**NR**	**NR**	**NR**	**30.8**	**109.1**	**34.9**	**58.2**	**65.7**	**167.2**
**Bill & Melinda Gates Foundation**	NR	NR	NR	NR	NR	NR	NR	NR	NR	NR	NR	NR	NR	NR	30.8	109.1	34.9	58.2	65.7	167.2
**GRAND TOTAL**	**38.3**	**0.1**	**72.6**	**0.0**	**75.4**	0.0	**155.6**	**0.5**	**219.3**	**1.1**	**187.0**	**1.9**	**368.5**	**0.5**	**542.3**	**110.9**	**717.1**	**60.2**	**2376.2**	**175.2**

Table 10. Search terms not found in any records (English, German, French)
The search terms below are listed under only one language, although many of the search terms were truncated specifically to ensure that they would identify the same or similar words in other languages. The wildcard “?” identifies any single character, while the wildcard “*" identifies an unlimited number of characters. The search was not case sensitive. Underscores are used below to represent blank spaces.

English	German	French
foetus	neugeboren	SMN
_foetal	totgeb	SMNE
_stillb	Wochenbett	mortinaissance
preterm	Malaria in der Schwangerschaft	mort-n
birthweight	Fr?hgeburt	poids de naissance
jaundice	unreif	faible poids
umbilic	niedriges Geburtsgewicht	jauniss
asphyxi	Untergewicht	_cord?n_
skin?to?skin	Lues connata	_allaite
Intermittent presumptive treatment	Ikterus	kangourou
Intermittent preventive treatment*pregnancy	Gelbsucht	peau???peau
IMNCI	Nabelinfektion	cordon ombilical
antenatal steroids	k?nguru	soins du cordon
_TT	frühes Stillen	PCIMNI
_amamant	Hautkontakt	anatoxin
	Nabelpflege	tétanique
	Laktat	Traitement pr?somptif intermittent*enceinte
	Malariatherapie in der Schwangerschaft	Traitement pr?ventif intermittent*enceinte
	Tetanusimpfung	Traitement pr?somptif intermittent*grossesse
		Traitement pr?ventif intermittent*grossesse
		tpi*grossesse
		tpi*enceinte

Table 11. Search terms not found in any records (Portuguese, Spanish, Italian, Dutch)
The search terms below are listed under only one language, although many of the search terms were truncated specifically to ensure that they would identify the same or similar words in other languages. The wildcard “?” identifies any single character, while the wildcard “*" identifies an unlimited number of characters. The search was not case sensitive. Underscores are used below to represent blank spaces.

Portugese	Spanish	Italian	Dutch
rec?m-nascido	_feto	natimortalit	pasgeboren
natimorto	mortinatalidad	nato mort	neonaat
nasce*morto	nacido muerto	malaria*gravidanza	foetaal
mal?ria durante a gravidez	nace* muerto	sottopeso	doodgebor
paludismo durante a gravidez	mortinato	sifilide	doogebor
mal?ria na gravidez	malaria durante el embarazo	itterizia	malaria in zwangerschap
paludismo na gravidez	paludismo durante el embarazo	ombelicale	prematuur
nascido muito cedo	peso de nacimiento	asfissia	geboorte gewicht
canguru	bajo peso	allatta	vroeg geboorte
_lactânc	icter?cia	pelle a pelle	navelstreng
Tratamento Intermitente Preventivo	canguro	lattazione	
umbigo	piel con piel	post-nat	
p?s-nata	Tratamiento preventivo intermitente*embarazo		
pele a pele	toxoide		
baixo peso ao nascimento			

Table 12. Titles and descriptions of misclassified records.

This presents an exhaustive list of all the records identified by our final list of search terms, which were subsequently coded as “misclassified” because the record was either wholly unrelated to newborn health or newborns were mentioned in some form of situation analysis while the funded activity clearly had no relevance to newborn health. Records with identical or near-identical descriptions have been aggregated, and the total number and value of records having the same description are listed in the notes.

#	Donor	Purpose code	Short description	project title	long description
Notes					
1	Italy	31165	AGRICULTURE AND FOOD SECURITY DISTRICT OF MUFINDI	Agriculture and food security district of Mufindi	Contribute to the reduction of HIV transmission from mothers to children during pregnancy, birth and breastfeeding to ensure proper care and treatment of viral reverse
$0.04m from 1 record: Two of the text fields and the purpose code indicate that this is an agriculture project and the long description appears totally unrelated.					
2	Switzerland	12220	COMPREHENSIVE CARE UNWANTED PREGNANCIES	Comprehensive Care Unwanted Pregnancies	Grossesses non désirées – soins aux jeunes femmes enceintes. Situation initiale: Plus d’un tiers des femmes ukrainiennes en âge de procréer sont stériles ou présentent des problèmes de fertilité. Dans 80 % des cas, c’est la conséquence d’avortements pratiqués de manière hasardeuse. Un programme de traitement médical des grossesses non désirées viendra donc s’ajouter aux programmes de médecine périnatale (médecine des nouveau-nés, obstétrique, etc.) et de promotion de la santé de la mère et de l’enfant (voir le projet numéro 7F-05620), déjà en place. Objectifs: La réglementation nationale et la formation médicale dans le domaine de l’avortement seront actualisées afin de tenir compte des connaissances les plus récentes. Pour mettre en oeuvre ce projet, l’organisation non gouvernementale (ONG) Women’s Health and Family Planning (WHFP) collabore étroitement avec l’OMS.
$0.2m from 2 records with identical descriptions: total value					
3	Germany	72040	EMERGENCY FOOD AID - EMOP 10559.0 - DELIVERY OF 209 MT VEGETABLE OIL AND 170 MT SUGAR	Emergency Food Aid - EMOP 10559.0 - Delivery of 209 mt Vegetable Oil and 170 mt Sugar	NMNH - EMOP 10559.0 - Lieferung von 209 mt Pflanzenöl und 170 mt Zucker
$39.8m from 35 disbursement records to many different countries. Other disbursements list different types and amounts of food. Germany has included the letters "MNH" as part of its project description of "NMNH".					
4	Germany	72010	EMERGENCY/DISTRESS RELIEF	NMNH / WEP - PRRO 10310.0, AUFSTOCKUNG DER PRRO SÜDLICHES AFRIKA, LIEFERUNG VON 1.984 T REIS VON ALGERIEN NACH SIMBABWE (TWINNING, ÜBERNAHME DER	
$0.7m from 1 record					

#	Donor	Purpose code	Short description	project title	long description
Notes					
				TRANSPORTKOSTEN)	
5	Germany	72010	EMERGENCY/DISTRESS RELIEF	NMNH FÜR KINDER UND SCHWANGERE/STILLENDE FRAUEN SOWIE REHABILITATIONSMAßNAHMEN (NEU: 2005.1823.3 / 687 20)	
$0.7m from 1 record					
6	Germany	72010	EMERGENCY/DISTRESS RELIEF	NMNH FÜR OPFER DES BÜRGERKRIEGES IN DARFUR, WEST SUDAN	
$1.1m from 1 record					
7	Spain	13020	IDENTIFICATION, PREVENTION AND ATTENTION OF OBSTETRIC FISTULAS AT KAYES REGION, REPUBLIC OF MALI	IDENTIFICATION, PREVENTION AND ATTENTION OF OBSTETRIC FISTULAS AT KAYES REGION, REPUBLIC OF MALI	EL TEMA CENTRAL DE ESTE PROYECTO ES LA LUCHA CONTRA LA FÍSTULA OBSTETRICIA. LA FÍSTULA OBSTETRICIA SE DEFINE MÉDICAMENTE COMO UNA COMUNICACIÓN ANORMAL ENTRE LA VEJIGA DE LA ORINA, EL ÚTERO Y LA VAGINA (A MENUDO TAMBIÉN ENTRE EL RECTO Y LA VAGINA) COMO CONSECUENCIA DE UN PARTO DISTÓCICO O ANORMAL. EL PARTO DISTÓCICO ES MUY FRECUENTE EN MALI, REPRESENTA DE 500 A 700 DEFUNCIONES MATERNAS POR CADA 100.000 PARTOS. LAS MUJERES MÁS AFECTADAS SON MADRES PRIMERIZAS, TIENEN ENTRE 13 Y 18 AÑOS Y VIVEN EN EL MUNDO RURAL. LA FÍSTULA OBSTETRICIA ES LA CONSECUENCIA DE UN PARTO PROLONGADO (UNA MEDIA DE 4 DÍAS) SIN INTERVENCIÓN MÉDICA, PERO TAMBIÉN ES EL RESULTADO DE UNA SITUACIÓN ECONÓMICA, POLÍTICA I CULTURAL: DESNUTRICIÓN, MATRIMONIOS PRECOCES, DESCONOCIMIENTO SOCIAL DE LAS FÍSTULAS, LA AUSENCIA DE ACOMPAÑAMIENTO EN EL EMBARAZO Y EL PARTO, INSUFICIENTE PERSONAL ESPECIALIZADO, MALA PRAXIS DE LOS PROFESIONALES POR FALTA DE PREPARACIÓN, ENTRE OTROS.LAS CONSECUENCIAS DE LA FÍSTULA SON FÍSICAS, PSÍQUICAS, SOCIALES Y ECONÓMICAS. ENTRE LAS SECUELAS DESTACAMOS LA INCONTINENCIA URINARIA Y/O FECAL, LA IMPOSIBILIDAD DE TENER HIJOS/AS Y DE LLEVAR UNA VIDA SEXUAL SATISFACTORIA. EN LA MAYORÍA DE LOS CASOS, EL RECIÉN NACIDO MUERE DURANTE EL PARTO. HAY QUE TENER EN CUENTA QUE, EN EL 90% DE LOS CASOS, LA LESIÓN SE PUEDE CURAR CON UNA SENCILLA INTERVENCIÓN QUIRÚRGICA (LA MAYORÍA DE LAS AFECTADAS DESCONOCE LA OPERACIÓN O NO LA PUEDEN PAGAR).UNA DE LAS CONSECUENCIAS MÁS RELEVANTES ES LA EXCLUSIÓN DE LA FAMILIA Y DE LA COMUNIDAD LO QUE REPRESENTA A SU VEZ EL AISLAMIENTO ECONÓMICO.LOS EJES CENTRALES DEL PROYECTO SON EL EMPODERAMIENTO DE LAS MUJERES Y EL FORTALECIMIENTO DE CAPACIDADES DEL PERSONAL SOCIO SANITARIO.LOS EJES DE ACCIÓN SON: ¿ DIVULGACIÓN E INFORMACIÓN SOBRE LA SALUD SEXUAL Y REPRODUCTIVA Y LAS FÍSTULAS
$0.3m from 1 record: This project mentions newborns, but only as a cause of obstetric fistula. The objectives of the project and the activities are about maternal health, not that of the newborn.					

#	Donor	Purpose code	Notes	Short description	project title	long description
						OBSTÉTRICAS.¿ APOYO A LAS MUJERES CON FÍSTULAS. ¿ FORMACIÓN DEL PERSONAL SOCIO SANITARIO.¿ ELABORACIÓN DE UN SISTEMA DE IDENTIFICACIÓN Y DE SEGUIMIENTO DE LOS CASOS DE FÍSTULAS EN LA REGIÓN PARA LA OBTENCIÓN DE DATOS FIABLES. ¿ ESTABLECIMIENTO DE RELACIONES INSTITUCIONALES EN EL ÁMBITO REGIONAL I NACIONAL.DE FORMA PARALELA SE REALIZA UN TRABAJO EN CATALUÑA CON MUJERES AGENTES DE SALUD DE MALÍ.
8	Spain	72010	$0.2m from 1 record: The title appears to be acidentally copied from another project, because the purpose code and long description indicate that it is a humanitarian aid project, and the recipient country and long description both indicate that the project is for Haiti, while the title mentions Morocco.	IMPROVE CHILD SURVIVAL	TOWARDS THE FULFILMENT OF THE MILLENIUM DEVELOPMENT GOAL NUMBER 4 IN MOROCCO: PROGRAMME FOR PERINATAL MORTALITY REDUCTION	Acción Humanitaria en Haití: Asegurar la supervivencia y protección de los niños en los próximos seis meses. Los esfuerzos se concentrarán en dar acceso a agua limpia y saneamiento, alimentación terapéutica, suministros médicos y refugios temporales.
9	Bill & Melinda Gates Foundation	12262	$0.1m from 1 record: "Fetal" is mentioned in this research project, but the aim of the project is not related to improving perinatal health.	MALARIA STOPPED BY A HUMAN PROTEIN THERAPEUTIC	Malaria Stopped by a Human Protein Therapeutic	to build on the recent discovery that elevated fetal hemoglobin (HbF), which alleviates sickle cell disease, can also confer malaria resistance
10	Sweden	43030	$0.3m from 1 record: The "IPTp" acronym is mentioned here in a completely different context, unrelated to malaria in pregnancy.	PORT ELIZABETH CONT. IPTP&TRAFFIC&KHULANI	Port Elizabeth Cont. IPTP&traffic&Khulani	3 year framework agreement. Capacity building and institutional strengthening within housing, transport, urban planning and urban environment. Also involves twinning with Gothenburg.
11	Spain	12182	$0.002m from 1 record: The purpose of this project is to study cocaine addiction using animal models.	MEDICAL RESEARCH	NEW THERAPEUTICAL STRATEGIES IN ADDICTION. PARTICIPATION OF ENDOCANNABINOID SYSTEM ON VULNERABIITY AND DEVELOPMENT OF COCAIN DEPENDENCY	Este proyecto pretende estudiar: 1) Las relaciones neuroanatómicas (inmunocitoquímica) y funcionales (PCR, Western Blotting) en las alteraciones conductuales provocadas por la adicción a cocaína en animales control sometidos a hipoxia perinatal; 2) El establecimiento de potenciales dianas famacológicas pertenecientes al sistema endocannabinoide para el restablecimiento de estos desórdenes comportamentales.

#	Donor	Purpose code	Short description	project title	long description
Notes					
12	Bill & Melinda Gates Foundation	12262	NOVEL MOUSE MODELS FOR TESTING HIV AND HCV VACCINES	Novel Mouse Models for Testing HIV and HCV Vaccines	The Balling consortium consists of a group of collaborating institutions each with specialized expertise and unique contributions to this ambitious, complex goal. The team has requested a supplement grant primarily to cover ongoing work at a key consortium institution, the Academic Medical Center at the University of Amsterdam (AMC). The AMC plays a unique and critical role in the project by providing the entire consortium with the tissues (adult and fetal human hematopoetic stem cells and hepatocytes) that are required to generate the humanized mice. The supplement funds would cover the higher-than-anticipated costs of the AMCÆs providing these critical reagents (i.e. humanized mice and associated tissues) to the members of the consortium, which broadly enable the projectÆs activities. In addition, the AMC team members are key members of the scientific leadership of the consortium, performing functional analyses of the mice created in the consortium; the supplement funds would cover the participation of these personnel for the remainder of the project period (through end of June 2010). The primary cause of the discrepancy between the original funding level and that required to meet the project goals is one inherent to early-stage scientific research: it simply took longer than expected to generate the required mouse strains for the project, due to the difficulties of breeding these delicate mice and to the need to re-derive some strains, a time-consuming and laborious process. In addition, it seems that during the original project planning and budgeting (now over 5 years ago), the critical role of the AMC to the projectÆs goals may have been underestimated. So while the original grant called for a phasing out of the AMC toward the end of the project, in reality AMC involvement will be necessary throughout the remainder of the project in order to complete the work and execute against project goals. to create novel mouse models for testing HIV and HCV vaccines
$2.0m from 3 records: Fetal tissues are used in the research "to generate the humanized mice" for HIV and HCV vaccines.					
13	Italy	2010	AGRICULTURE AND FOOD SECURITY DISTRICT OF MUFINDI	Agriculture and food security district of Mufindi	Contribute to the reduction of HIV transmission from mothers to children during pregnancy, birth and breastfeeding to ensure proper care and treatment of viral reverse
$0.04m from 1 record: The long description, which mentions relevant activities, seems to be mistakenly transferred from another project, as the purpose code, title, and short description all indicate that this is an agriculture project and there is no indication of a connection between the long description and agriculture.					

Table 13. Titles and descriptions of non-research projects exclusively benefitting newborns.
This is an exhaustive list of the record descriptions categorised as non-research projects exclusively benefitting newborns. The 59 sets of descriptions here reflect 430 different records, as many records include identical descriptions. In a few cases, projects for which the title, short description, and long description were virtually identical have been included as a single entry. For example, the U.S. provided many projects with information identical to row 28, except that the implementing organization listed at the end of the long description varied.

	project title	Short description	long description
1	10733. Antibiotics to treat sepsis of the newborn	ANTIBIOTICS TO TREAT SEPSIS OF THE NEWBORN	Antibiotics to treat sepsis of the newborn
2	APPUI AUX FORMATIONS EN ÉCHOGRAPHIE PÉRINATALE	APPUI AUX FORMATIONS EN ÉCHOGRAPHIE PÉRINATALE	APPUI AUX FORMATIONS EN ÉCHOGRAPHIE PÉRINATALE
3	Human milk banking for Lima Department. Peru	BASIC NUTRITION	Este proyecto llevado a cabo con la participación del Instituto Nacional Materno Perinatal en Lima y que se ha decidido subvencionar tendrá como objetivo principal, disminuir la morbilidad y mortalidad de los recién nacidos asociada a la falta de alimentación de leche materna, mediante la creación de un Banco de Leche institucional.
4	[blank]	BASIC HEALTH CARE	Conference support activities for international conference on newborn hearing loss. Contract awarded to ARSI, an Italian organization.
5	Registration Process of Newborns	BASIC HEALTH INFRASTRUCTURE	Registration Process of Newborns
6	WHO NT ELIMINATION & SAFE EPI INJECTIONS	BASIC HEALTH: WHO NT ELIMINATION & SAFE EPI INJECTIONS	The 3-year $3 million project commenced late in 1998-99. The project aims to maintain the drive of Neonatal Tetanus (NT) elimination as part of the national campaign and achieve the goals of ensuring 100% safe Expanded Program of Immunisation (EPI) injec
7	CAP. FIRST STAGE OF IMPROVEMENT ON PRE-NATAL CARE QUALITY	CAP. FIRST STAGE OF IMPROVEMENT ON PRE-NATAL CARE QUALITY	EL PROYECTO PRETENDE LA PROVISIÓN DE EQUIPAMIENTO E INSUMOS PARA ASISTIR EN CONDICIONES ÓPTIMAS A 1.600 RECIÉN NACIDOS, CONTRIBUYENDO CON ELLO AL DESCENSO DE LA MORTALIDAD NEONATAL HOSPITALARIA EN LA PROVINCIA DE TUCUMÁN.
8	CAP. IMPROVEMENT ON PERINATAL HEALTH CARE IN SAN MIGUEL REGION IN TUCUMÁN	CAP. IMPROVEMENT ON PERINATAL HEALTH CARE IN SAN MIGUEL REGION, TUCUMÁN	SUBVENCIÓN DE CONVOCATORIA ABIERTA Y PERMANENTE A LA ONG MENSAJEROS DE LA PAZ PARA EL PROYECTO MEJORA DE LA CALIDAD DE ATENCIÓN SANITARIA PERINATAL DE LA ZONA DEL GRAN SAN MIGUEL DE TUCUMÁN (ARGENTINA).
9	AYUDA A RECIÉN NACIDOS EN GRAVE RIESGO Y AYUDA MALTESA ARGENTINA PARA LA PREVENCIÓN DEL SIDA PERINATAL	EMERGENCY/DISTRESS RELIEF	AYUDA A RECIEN NACIDOS EN GRAVE RIESGO Y AYUDA MALTESA ARGENTINA PARA LA PREVENCIÓN DEL SIDA PERINATAL
10	EXPANSION OF ESSENTIAL NEWBORN CARE	EXPANSION OF ESSENTIAL NEWBORN CARE	THIS PROJECT AIMS TO STRENGTHEN THE CAPACITY IN REPRODUCTIVE HEALTH BY PROVIDING TECHNICAL ASSISTANCE TO PROVIDERS, BY ENHANCING AWARENESS RAINSING AND BY PROMOTING KEY FAMILY AND COMMUNITY PRACTICES
11	Global Neonatal Survival Initiative	GLOBAL NEONATAL SURVIVAL INITIATIVE	to reduce neonatal deaths in developing countries by focusing on increasing global and national attention, strengthening policies and programs, and improving technologies and approaches available to address this critical need
12	DOTACIÓN DE EQUIPO SERVICIO DE NEONATOLOGÍA HOSPITAL 11 DE MAYO - MATERNIDAD	GRANT OF NEONATOLOGY DEPARTMENT EQUIPMENT, 11 OF MAY HOSPITAL/MATERNITY	DOTACIÓN DEL SERVICIO DE NEONATOLOGÍA HOSPITAL 11 DE MAYO - MATERNIDAD
13	THE PROJECT FOR EXPANSION OF IMMUNIZATION AGAINST NEONATAL TETANUS	EXPANSION OF IMMUNISATION AGAINST NEONATAL TETANUS	[blank]

14	Fredskorpset personnel exchange	FREDSKORPSET PERSONNEL EXCHANGE	Change knowledge, attitudes and practice among in the hospitals in relation to newborn care in general, and the care for sick newborn in especially.
15	IMPROVEMENT OF COMPREHENSIVE HEALTH CARE OF NEONATOLOGY WITHIN THE HOSPITAL HERNANDEZ VERA - SANTA CRUZ DE LA SIERRA (2009) (44.822,00) (TOTAL 44.822,00)	IMPROVEMENT OF COMPREHENSIVE HEALTH CARE OF NEONATOLOGY WITHIN THE HOSPITAL HERNANDEZ VERA - SANTA CRUZ DE LA SIERRA (2009) (44.822,00) (TOTAL 44.822,	LA FUNCIÓN ES MEJORAR LA SALUD DE LOS BEBÉS QUE NACEN CON INSUFICIENCIA RESPIRATORIA, SIENDO ACTUALMENTE UNO DE LOS SINTOMAS MÁS FRECUENTES QUE MUESTRAN EN LOS PRIMEROS MOMENTOS DE SU VIDA. A TRAVÉS DEL RESPIRADOR SE LE AYUDA A LOS BEBÉS RECÉN NACIDOS A RESPIRAR PROPORCIONÁNDOLE POR UNA PARTE OXIGENO Y AIRE RESPIRABLE EN DISTINTA PROPORCIÓN,
16	INTRODUCING SOUND INFANT CARE SYSTEMS IN LAO PDR	INTRODUCING SOUND INFANT CARE SYSTEMS IN LAO PDR	THE AIM IS TO IMPROVE MATERNAL AND CHILD HEALTH IN LAO PDR BY STRENGHTENING THE EXISTING NEONATAL CARE NETWORK - PROV. SALAVAN, SEKONG AND ATTAPEU
17	Loterie Nationale -- MDG's Réduire la mortalité périnatale, projet Casa de Espera'	LOTERIE NATIONALE -- MDG'S RÉDUIRE LA MORTALITÉ PÉRINATALE, PROJET CASA DE ESPERA'	Loterie Nationale -- MDG's Réduire la mortalité périnatale, projet Casa de Espera'
18	MEDICAL CARE FOR NEWBORN CHILDREN WITH BIRTH PROBLEMS IN SECURE ENVIRONM	MEDICAL CARE FOR NEWBORN CHILDREN WITH BIRTH PROBLEMS IN SECURE ENVIRONM	MEDICAL CARE FOR NEWBORN CHILDREN WITH BIRTH PROBLEMS IN SECURE ENVIRONMENT AT THE MATERNITY HOSPITAL OF THE IRAQUI AL AMAL ORGANIZATION IN ERBIL. PROVISION OF EQUIPMENT FOR THE NEWBORN INTENSIVE CARE UNIT - STERILIZATION EQUIPMENT FOR HOSPITAL'S SURGERIE
19	Tashkent perintal centre project	MEDICAL SERVICES	Tashkent perintal centre project, refitting The Perinatal Center's departments with modern furniture, equipment and instruments. Improveing the service standard into international level.
20	Modernising Perinatology	MODERNISING PERINATOLOGY	Modernising Perinatology
21	SEGUIMIENTO DE RECIÉN NACIDOS DE ALTO RIESGO - SERENAR	MONITORING NEW BORNS AT HIGH RISK - SERENAR	MEJORAR LA PREVENCIÓN DETECCIÓN Y ATENCIÓN TEMPRANA DE NIÑOS NACIDOS EN SITUACIÓN DE RIESGO EN SU DESARROLLO PSICO-NEURO-SENSORIAL EN INSTITUCIONES DE SALUD PÚBLICA URUGUAYAS A TRAVÉS DE LA CREACIÓN DE UNIDADES DE ATENCIÓN TEMPRANA EN LOS DEPARTAMENTOS D
22	Éducation et formation plurisectorielles	MULTISECTOR EDUCATION/TRAINING	colloque L. Brutus Conséquences de la maladie de Chagas au cours de la grossesse pour le nouveau-né. Traitement de la maladie de Chagas congénitale
23	[blank]	MULTISECTOR EDUCATION/TRAINING	Attrezzature mediche minime per il monitoraggio e la cura del neonato, formazione personale sanitario, sensibilizzazione comunità trentina
24	DEVELOPMENT AND IMPLEMENTATION OF NEONATAL HEALTH ACTIVITIES	NEONATAL HEALTH ACTIVITIES	Provision of Funds to meet the cost of advice, commisioning projects, monitoring to develop HPD child Health team's activities in Neonatal Health
25	[blank]	NEONATAL IMMUNISATION WITH PNEUMOCOCCAL CONJUGATE VACCINE	[blank]
26	NEONATOLOGY IN UKRAINE	NEONATOLOGY IN UKRAINE	NEONATOLOGY IN UKRAINE
27	Newborn care	NEWBORN CARE	Newborn care
28	Newborn Care and Treatment	NEWBORN CARE AND TREATMENT	Improve care of male and female newborns by identifying and treating serious infections and other neonatal complications, including immediate care at birth (resuscitation, thermal stabilization, cord care, breastfeeding), the provision of routine essential care, special care for low birth weight and premature infants, increasing family and health worker recognition of newborn danger signs, and increasing access to appropriate treatment when required. [Enterprise - sometimes specified on individual records]
29	4035. Newborn care in community	NEWBORN CARE IN COMMUNITY	Newborn care in community
30	NORWAC. Perinatal care	NORWAC. PERINATAL CARE	Perinatal care for women and children in Chair Special Gynecologiacal Hospital
31	Ospedali periferici di Timor Est	OSPEDALI PERIFERICI DI TIMOR EST	Creation of five new neonatal intensive care units at the peripheral hospitals in East Timor

32	PCI. Congenital hypothyroidism diagnosis in Nicaragua: creation of the National Neonatal Screening Center	PCI. CONGENITAL HYPOTHYROIDISM DIAGNOSIS IN NICARAGUA: CREATION OF THE NATIONAL NEONATAL SCREENING CENTER	DIAGNÓSTICO DEL HIPOTIROIIDISMO CONGÉNITO EN NICARAGUA: CREACIÓN DEL CENTRO NACIONAL DE CRIBADO NEONATAL
33	Audiovisual program for creation of didactic units for teachers training and health collaborators in neonatology (resuscitation and basic care for ne	PERSONNEL DVPT: POP. & REPRO HEALTH	Creación de Unidades didácticas para la formación de personal sanitario de primera línea mediante cápsulas audiovisuales que muestren, de una manera sencilla, los principios fundamentales en las técnicas de reanimación y cuidados básicos del recién nacido. Además se pretende das formación básica al personal sanitario mozambiqueño en el manejo de la Web TV de la plataforma de Divulgación Científica de la UCM para compartir, mediante la misma, información básica y establecer un seminario on-line permanente para la mejora formativa en técnicas de neonatología y ecografía a distancia.
34	Pilot program for child blindness prevention	PILOT PROGRAM FOR CHILD BLINDNESS PREVENTION	La retinopatía del prematuro es la principal causa de ceguera infantil. Esta patología se está presentando en Sudamérica y especialmente en Bolivia con una frecuencia exponencial en los últimos años debido a la instauración de unidades neonatales, capaces de salvar la vida de muchos prematuros pero dejándolos con gravísimas secuelas visuales al no poder facilitarles los tratamientos precisos para prevenir y tratar esta enfermedad. Tratando precozmente la retina de los bebés afectados se puede reducir gran parte de las secuelas de la Retinopatía del prematuro. Por ello el proyecto pretende establecer un programa piloto de prevención y tratamiento de la enfermedad en las tres principales maternidades de la zona y que sirva de modelo para su establecimiento en otras zonas de Bolivia y países del entorno.
35	COSTRUZIONE DI UN CENTRO NEONATALE PER MONITORARE LA SALUTE DEI BAMBINI E ORFANOTROFIO PER BAMBINI ORFANI	POPULATION POLICY AND ADMIN. MGMT	[blank]
36	Neonatal immunisation with pneumococcal conjugate	POPULATION POLICY AND ADMIN. MGMT	[blank]
37	MONITORING OF THE RECENTLY BORN IN SITUATION OF	REPRODUCTIVE HEALTH CARE	SEGUIMIENTO DE RECIÉN NACIDOS DE ALTO RIESGO
38	PREDICTION OF PERINATAL PREMATURITY BECAUSE OF THE ULTRASOUND TRANSMISSION TECHNIQUE	PREDICTION OF PERINATAL PREMATURITY BECAUSE OF THE ULTRASOUND TRANSMISSION TECHNIQUE	PREDICCIÓN DE PREMATURIDAD PERINATAL POR LA TÉCNICA DE TRANSMISIÓN ULTRASÓNICA (PRÉDICTION D'UN ACCOUCHEMENT PRÉMATURÉ PAR LA TECHNIQUE DE TRANSMISSION ULTRASONIQUE)
39	SUPPORT FOR THE HOSPITAL OF KILAMBA KIAXI TO IMPROVE CHILD HEALTH CARE	REPRODUCTIVE HEALTH CARE	APOYO AL HOSPITAL DE KILAMBA KIAXI PARA MEJORAR LA ATENCIÓN SANITARIA A LOS NEONATOS
40	Prévention, dépistage précoce et prise en charge des invalidités du nouveau-né	PRÉVENTION, DÉPISTAGE PRÉCOCE ET PRISE EN CHARGE DES INVALIDITÉS DU NOUVEAU-NÉ	Prévention, dépistage précoce et prise en charge des invalidités du nouveau-né
41	[blank]	REPRODUCTIVE HEALTH CARE	To: (a) identify urban and rural communities in South Africa with high proportions of childbearing-aged women who are at risk for an alcohol exposed pregnancy that could result in Fetal Alcohol Syndrome (FAS); and (b) to develop a model prevention program aimed at reducing hazardous alcohol use and/or promoting pregnancy delay until alcohol abuse is resolved in those women at highest risk.
42	PROG. DE LUTTE/MORTALITE NEONATALE ET PREVENTION DU HANDICAP	PROG. DE LUTTE/MORTALITE NEONATALE ET PREVENTION DU HANDICAP	[blank]
43	THE PROJECT FOR EXPANSION OF IMMUNIZATION AGAINST NEOMATAL TETANUS	PROJECT FOR EXPANSION OF IMMUNIZATION AGAINST NEONATAL TETANUS	[blank]
44	THE PROJECT FOR NEONATAL TETANUS CONTROL	PROJECT FOR NEONATAL TETANUS CONTROL	[blank]

45	REDUCTION OF NEONATAL MORBIDITY AND MORTALITY IN THE DOMINICAN REPUBLIC	REDUCTION OF NEONATAL MORBIDITY AND MORTALITY IN THE DOMINICAN REPUBLIC	LA CAPACITACIÓN EN URGENCIAS NEONATALES A 100 PROFESIONALES DE ENFERMERÍA (35 ENFERMERAS/OS Y 63 AUXILIARES DE ENFERMERÍA) DEL PRIMER Y SEGUNDO NIVEL DE SALUD QUE LABORAN EN LA REGIONES SANITARIA 0 DE REPÚBLICA DOMINICANA QUE OBTENDRÁN CON LA FORMACIÓN TEÓRICO - PRÁCTICA. LA DOTACIÓN DEL HOSPITAL BENEFICIARIO DEL PROYECTO CON MATERIAL DE ALTO NIVEL DE ESPECIALIZACIÓN
46	Reduction of Neonatal Mortality by Upgrading Escuela Hospital	REDUCTION OF NEONATAL MORTALITY BY UPGRADING ESCUELA HOSPITAL	[blank]
47	Smoking Cessation Intervention for Disadvantaged Pregnant Women (South Africa) Phase II	SMOKING CESSATION INTERVENTION FOR DISADVANTAGED PREGNANT WOMEN (SOUTH AFRICA) PHASE II	To develop and evaluate a smoking cessation intervention for disadvantaged, coloured pregnant women for the purposes of increasing quit rates and thereby improving perinatal outcomes among this group of women.
48	Renewal of equipment for neonatal intensive care unit of the hospital Jesus Mary Joseph	RENEWAL OF EQUIPMENT FOR NEONATAL INTENSIVE CARE UNIT OF THE HOSPITAL JESUS MARY JOSEPH	Renewal of equipment for neonatal intensive care unit of the hospital Jesus Mary Joseph
49	IMPROVING PERINATAL CARE TO REDUCE INFANT AND CHILD MORTALITY IN POOR COUNTRIES	REPROD. HEALTH CARE: CHILD HEALTH KNOWLEDGE PROG.	To inform policy and practice and influence climate of opinion to help ensure better outcomes on key topics within perinatal care relevant to the health of poor people
50	KP14 Child Health Knowl'Prog	REPRODUCTIVE HEALTH CARE	To inform policy and practice and influence climate of opinion to help ensure better outcomes on key topics within perinatal care relevant to the health of poor people
51	Prévention, dépistage précoce et prise en charge des invalidités du nouveau-né	SOCIAL/WELFARE SERVICES	Prévention, dépistage précoce et prise en charge des invalidités du nouveau-né
52	Regional Proverty Alleviation Programme : Rural Community Development-Ph	REPRODUCTIVE HEALTH CARE	Remodeling the neonatal care unit of the hospital Escuela and providing
53	TOWARDS THE FULFILMENT OF THE MILLENIUM DEVELOPMENT GOAL NUMBER 4 IN MOROCCO: PROGRAMME FOR PERINATAL MORTALITY REDUCTION	TOWARDS THE FULFILMENT OF THE MILLENIUM DEVELOPMENT GOAL NUMBER 4 IN MOROCCO: PROGRAMME FOR PERINATAL MORTALITY REDUCTION	HACIA EL LOGRO DEL ODM 4 EN MARRUECOS PROGRAMA PARA LA REDUCCIÓN EN LA MORTALIDAD NEONATAL - SE PRETENDE REDUCIR LA MORTALIDAD NEONATAL PROPORCIONANDO SERVICIOS DE SALUD Y COMITÉS PROVINCIALES DE CONTROL NEONATAL REALIZANDO CURSOS DE FORMACIÓN PARA PREVENIR ESTE PROBLEMA.
54	TRAINING ON PEDIATRICS-NEONATOLOGY: NEW BORNS ASSITANCE IN DELIVERY ROOM. FEEDING, AND MALNUTRITION AND VERTICAL TRANSMISSION PREVENTION. CARES IN ELEMENTARY ATTENTION	TRAINING ON PEDIATRICS-NEONATOLOGY: NEW BORNS ASSITANCE IN DELIVERY ROOM. FEEDING, AND MALNUTRITION AND VERTICAL TRANSMISSION PREVENTION. CARES IN ELE	FORMACIÓN DE PERSONAL SANITARIO PARA MEJORAR LA ATENCIÓN A LOS RECIÉN NACIDOS EN LA SALA DE PARTOS, PREVENCIÓN DE LAS ENFERMEDADES DE TRANSMISIÓN VERTICAL Y ALIMENTACIÓN AL PRIMER MES DE VIDA, Y DE LA MALNUTRICIÓN, ASÍ COMO PRIMAR LA IMPORTANCIA DE REALIZACIÓN DE EXÁMENES DE SALUD Y VACUNACIÓN EN LA ATENCIÓN PRIMARIA.
55	SUPPORT FOR UNICEF IN ERITREA	SUPPORT FOR UNICEF IN ERITREA	REDUCCIÓN DE LA MORTALIDAD NEONATAL HACIA EL CUMPLIMIENTO DEL 4º OBJETIVO DE DESARROLLO DEL MILENIO: PROPORCIONAR EL ACCESO A LA GESTIÓN INTEGRADA DE CALIDAD EN EL HOGAR Y FACILITAR LA IMPLEMENTACIÓN DE SERVICIOS PARA ENFERMEDADES NEONATALES E INFANTILES A UN 60% DE LOS RECIÉN NACIDOS Y NIÑOS MENORES DE CINCO AÑOS EN SUB-REGIONES (ZOBAS) DE GASHA BARKA Y MARKEL.
56	Tashkent perintal centre project	TASHKENT PERINTAL CENTRE PROJECT	Refitting The Perinatal Center_s departments with modern furniture_ equipment and instruments. Improving the service standard into international level
57	THE PRIJECT FOR EXPANSION OF IMMUNIZATION AGAINST NEONATAL TENTANUS	THE PRIJECT FOR EXPANSION OF IMMUNIZATION AGAINST NEONATAL TENTANUS	[blank]
58	To reduce newborn mortality through training of neonatologists as trainers in respiratory disorders of the newborn; evaluate implementation	TO REDUCE NEWBORN MORTALITY THROUGH TRAINING OF NEONATOLOGISTS AS TRAINERS IN RESPIRATORY DISORDERS OF THE NEWBORN; EVALUATE IMPLEMENTATION	To reduce newborn mortality through training of neonatologists as trainers in respiratory disorders of the newborn; evaluate implementation in clinical practice of the training in use of neonatal equipment provided in 2008.

59	Construction de néonatologie du Holy Family Hospital de Bethlehem	CONSTRUCTION DE NÉONATOLOGIE DU HOLY FAMILY HOSPITAL DE BETHLEHEM	Bouw van de afdeling neonatologie in het Holy Family Hospital

Table 14. Titles and descriptions of research activities mentioning newborns but also benefiting other population groups.
This is an exhaustive list of the record descriptions categorised as research projects mentioning newborns but also benefitting other population groups. The 48 sets of descriptions here reflect 62 different records, as many records include identical descriptions. In a few cases, projects for which the title, short description, and long description were virtually identical have been included as a single entry. For example, the World Health Organization provided four projects with information identical to row 10, except that the region listed at the end of the long description varied.

	project title	Short description	long description
1	South Africa Programme Managed at Pretoria - HIV/AIDS - WITS - PHRU - WITS PERINATAL HIV RESEAR	BASIC HEALTH CARE	South Africa Programme Managed at Pretoria - HIV/AIDS - WITS - PHRU - WITS PERINATAL HIV RESEAR
2	BEGINNING OF A PROGRAM TO PREVENT MENTAL DISABILITY FROM CONGENITAL HYPOTHYROIDISM AND DIAGNOSIS OF THE CHAGAS DISEASE	MEDICAL RESEARCH	INICIACIÓN DE UN PROGRAMA DE PREVENCIÓN DE LA DISCAPACIDAD MENTAL POR HIPOTIROIDISMO CONGÉNITO NEONATAL Y DIAGNÓSTICO SEROLÓGICO DE LA ENFREMEDAD DE CHAGAS
3	EXTENSION TO NICARAGUA OF THE PROGRAM FOR THE PREVENTION OF MENTAL DISABILITY ASSOCIATED WITH NEONATAL CONGENITAL HYPOTHYROIDISM	MEDICAL RESEARCH	EXTENSIÓN A NICARAGUA DEL PROGRAMA DE PREVENCIÓN DE LA DISCAPACIDAD MENTAL ASOCIADA AL HIPOTIROIDISMO CONGÉNITO NEONATAL
4	[blank]	MEDICAL RESEARCH	Newborn screening is recognized as an essential, life-saving and effective preventive public health service that tests babies for congenital disorders that are not apparent at birth. If these disorders are not quickly detected and treated, they cause mental retardation, severe illness, or premature death. The CDC Newborn Screening Quality Assurance Program has the only laboratory in the world devoted to ensuring the accuracy of newborn screening tests. NSQAP provides quality control (QC) materia
5	A new way to prevent HIV infection during breastfeeding.	A NEW WAY TO PREVENT HIV INFECTION DURING BREASTFEEDING.	We have invented a novel, low-cost, modification of a conventional nipple shield to be discretely used by HIV+ mothers to prevent MTCT during breasifeeding (patent pending). During the passage of breast milk through the shield, HIV will be killed by contact with the treated filter. The device could also be a new drug delivery method. to prevent HIV infection during breastfeeding
6	Accessible Quality-Assured Diagnostic Tests for Sexually Transmitted Infections	ACCESSIBLE QUALITY-ASSURED DIAGNOSTIC TESTS FOR SEXUALLY TRANSMITTED INFECTIONS	To reduce the burden of sexually transmitted infections (STIs) and their complications in the developing world by increasing access to STI diagnostic testing in pregnant women and those at high risk of acquiring and transmitting STIs, and by developing tools to ensure the quality of the STI tests and testing in resource-limited settings. Funds requested for this project will be used to demonstrate the attributable benefit of increased access to STI diagnostics in the developing world and to develop a framework and tools for the introduction and sustainable adoption of quality-assured rapid tests for sexually transmitted infections in resource-limited settings. to produce a global framework and tools for the introduction and sustainable adoption of quality-assured rapid bacterial sexually transmitted infection (STI) tests. In order to provide evidence of feasibility of test introduction and cost-effectiveness, demonstration projects will be conducted to validate the attributable benefit of increased access to rapid quality-assured STI tests in two populations for which access to diagnostics are urgent priorities, syphilis screening for pregnant women and STI screening for high risk populations.
7	Alive & Thrive	ALIVE & THRIVE	to develop and evaluate models for delivering integrated breastfeeding & complementary feeding interventions at scale in Bangladesh, Ethiopia, Vietnam and disseminate lessons for global adoption.

	project title	Short description	long description
8	Breastmilk shield to prevent HIV transmission.	BREASTMILK SHIELD TO PREVENT HIV TRANSMISSION.	to study the efficacy of using newly developed copper-oxide based filters that deactivate a wide range of viruses, including HIV-1, as a shield to enable HIV-infected mothers to breastfeed their infants without risking transmission of the virus
9	SIFILIS CONGENITA EN EL DEPARTAMENTO CAPITAL DE MISIONES (ARGENTINA). ESTUDIO EPIDEMIOLOGICO CON INTERVENCIONES DE PREVENCION Y PROMOCION A MUJERES EN RIESGO.	CONGENITAL SYPHILIS IN THE DEPARTMENTO CAPITAL DE MISIONES (ARGENTINA). EPIDEMIOLOGICAL STUDY WITH P	CONGENITAL SYPHILIS IN THE DEPARTMENTO CAPITAL DE MISIONES (ARGENTINA). EPIDEMIOLOGICAL STUDY WITH PREVENTION AND PROMOTION INTERVENTIONS FOR WOMEN AT RISK .
10	CVCA. Health research capacity	CVCA. HEALTH RESEARCH CAPACITY	Reproductive health care, including maternal health, Newborn and child health: [region]
11	GCE: Maternal Immunization to protect infants against malaria	GCE: MATERNAL IMMUNIZATION TO PROTECT INFANTS AGAINST MALARIA	The idea is a needle-free malaria vaccine administered to young women of child-bearing age before pregnancy and boosted after delivery to induce protective IgA and IgG antibodies against malaria in breast milk. A related idea is to administer the vaccine to a nursing mother so that the vaccine appears in breast-milk; tweaking the babyÆs immune system allows the infant immune system to respond to the antigen in breast milk and thereby resulting in active immunity against malaria. An approach to immunize mothers to confer and induce antimalarial immunity in babies through breastfeeding
12	Going to Scale: Policy Change and Research for Emergency Obstetric and Newborn Care	GOING TO SCALE: POLICY CHANGE AND RESEARCH FOR EMERGENCY OBSTETRIC AND NEWBORN CARE	Funding from the Gates Foundation will support activities designed to meet two objectives: (1) Catalyze policy and funding processes at international and national levels to increase equitable and affordable access to emergency obstetric and newborn care; and (2) Generate new knowledge critical for meeting the MDGs on maternal health and child mortality. Expected outcomes are an increase in policies and programs that enhance equity and access to emergency obstetric and newborn care, and research findings that contribute to the knowledge base on expanding equitable access to emergency obstetric and newborn care, and strengthening health systems. All activities will be done collaboratively, with policy, research and program colleagues in national governments, UN agencies and NGOs. Gates Foundation funds will support Columbia University staff, consultants and sub-contractors situated around the globe; travel costs associated with the collaborative policy, strategy and research activities; and administrative costs. to identify and develop policies and strategies that will enable countries to deliver critical maternal and neonatal mortality interventions
13	PIC 2008 Etude épidémiologique de la carence et de la surcharge en iode	HIGHER EDUCATION	CIUF PIC 2008 Etude épidémiologique de la carence et de la surcharge en iode, impact sur les altérations de la fonction thyroïdienne maternelle et néonatale à Lubumbashi et développement d'une stratégie de contrôle - PIC 2008 Etude épidémiologique de la carence et de la surcharge en iode - L¿objectif global de ce projet est d¿améliorer l¿état de santé du couple mère-enfant à Lubumbashi par un meilleur suivi de la fonction thyroïdienne. Plus spécifiquement on y étudiera le profil épidémiologique de la carence et de la surcharge en iode et le - Ce projet sur le dépistage précoce de la carence iodée, de la surcharge en iode et des altérations de la fonction thyroïdienne maternelle et néonatale permettra de rendre disponible et opérationnel à Lubumbashi une Unité d¿endocrinologie et renforcer les
14	ICDDRB 2010-2011 Coresupp	ICDDRB 2010-2011 CORESUPP	The International Centre for Diarrhoeal Disease Research, Bangladesh - is an international health research institution located in Dhaka dedicated to saving lives through research and treatment addressing some of the most critical health concerns facing the world today, ranging from improving neonatal survival to HIV/AIDS. This is the core support provided by Sida's team in Dhaka.
15	Identify opportunities to test scalable models that more effectively reach pregnant women, women during delivery, and newborns in	IDENTIFY OPPORTUNITIES TO TEST SCALABLE MODELS THAT MORE EFFECTIVELY REACH PREGNANT WOMEN.	Identify opportunities to test scalable models that more effectively reach pregnant women, women during delivery, and newborns in Northern Nigeria. (This will include scoping of demand, practice, and care-seeking during critical times, alternative linkages between homes/communities

	project title	Short description	long description
	homes in Northern Nigeria		and skilled providers during delivery and for illness, and the potential for market-based solutions). to identify opportunities to test scalable models that more effectively reach pregnant women, women during delivery, and newborns in homes in Northern Nigeria. Building on linkages between homes/communities and a range of alternative private providers, improve care seeking for illness and home based practices.
16	Improving maternal, neonatal and child health outcomes through better designed nutrition policies and programs	IMPROVING MATERNAL, NEONATAL AND CHILD HEALTH OUTCOMES THROUGH BETTER DESIGNED NUTRITION POLICIES AND PROGRAMS	Our project goal is to improve maternal, neonatal, and child health (MNCH) outcomes through better designed policies and programs that enhance nutrition throughout the life cycle, with a particular focus on maternal nutrition. We will bring together a multidisciplinary group of experts to identify existing and new interventions with the potential to improve our health outcomes of interest and to determine how these could be implemented more effectively in resource-poor settings. We will identify priority research needed to bolster the evidence for promising new interventions as well as operations research needed to overcome demand and supply barriers to implementation of current and new strategies and interventions. The project will begin January 1, 2010 and last for 18 months. to develop priorities for a maternal and fetal nutrition research and implementation agenda
17	IMT - Centre MURAZ Santé Maternelle et Nouveau-nés, Paludisme, Nutrition	IMT - CENTRE MURAZ SANTÉ MATERNELLE ET NOUVEAU-NÉS, PALUDISME, NUTRITION	ITG - Centre MURAZ / Santé maternelle et nouveau-nés, paludisme, nutrition
18	CONGENITAL SYPHILIS EN THE DEPARTMENT HEADQUATERS OF MISIONS (ARGENTINA). INVESTIGATION ON EPIDEMIOLOGY WITH INTERVENTIONS OF PREVENTION AND PROMOTION TO WOMEN IN RISK.	INFECTIOUS DISEASE CONTROL	SE TRATA DE CONTRIBUIR A DISMINUIR LA INCIDENCIA DE LA SÍFILIS CONGÉNITA MEDIANTE INVESTIGACIÓN DESCRIPTIVA Y ANALÍTICA PARA CONOCER LOS FACTORES ASOCIADOS Y POSTERIORMENTE CAPACITAR A LOS RECURSOS HUMANOS DE ATENCIÓN PRIMARIA PARA PROMOVER LA SALUD SEXUAL Y REPRODUCTIVA
19	Innovative solutions to critical implementation bottlenecks for maternal, neonatal and child health	INNOVATIVE SOLUTIONS TO CRITICAL IMPLEMENTATION BOTTLENECKS FOR MATERNAL, NEONATAL AND CHILD HEALTH	to identify and test innovative solutions to critical implementation bottlenecks in priority countries focusing on increasing coverage of effective interventions for maternal neonatal and child health
20	INSTITUTIONAL SUPPORT TO ICDDR,B - PROJECT EVALUATION AND DESIGN	INSTITUTIONAL SUPPORT TO ICDDR	ADDITIONAL SECTORS: 012220 (10%), 012240 (10%), 012250 (10%), 012282 (10%), 013020 (10%), 013030 (10%), 013040 (10%) EXPECTED IMPACT / LONG-TERM RESULTS: IMPROVED HEALTH CONDITIONS FOR THE PEOPLE OF BANGLADESH THROUGH STRENGTHENING THE INSTITUTIONAL CAPACITY OF ICDDR,B TO MEET ITS OBJECTIVES EXPECTED OUTCOMES / MEDIUM-TERM RESULTS: CONTRIBUTING TO THE INTRODUCTION OF COST-EFFECTIVE STRATEGIES FOR ZINC THERAPY IN DIARRHOEA HELPING TO REDUCE MATERNAL MORBIDITY AND MORTALITY AND IMPROVING PERINATAL AND NEONATAL HEALTH DEVELOPING AN EFFECTIVE PACKAGE FOR THE PREVENTION OF FOETAL GROWTH RESTRICTION HELPING TO IDENTIFY A PACKAGE OF SUITABLE VACCINES FOR DIARRHOEA AND ACUTE RESPIRATORY INFECTIONS DEFINING THE BURDEN OF TUBERCULOSIS AND IDENTIFYING EFFECTIVE STRATEGIES FOR PREVENTION AND CONTROL ADDRESSING THE STAGNATION OF FERTILITY DECLINE HELPING TO PREVENT THE EPIDEMIC OF HIV/AIDS AND RTI/STI CONTRIBUTING TO KNOWLEDGE THAT CAN IMPACT THE BURDEN OF VECTOR-BORNE DISEASES EXPECTED OUTPUTS / SHORT-TERM RESULTS: TO BE DETERMINED
21	Integrating FP Best Practices in Pakistan	INTEGRATING FP BEST PRACTICES IN PAKISTAN	This is a $496,000 proposal to promote the implementation of high-impact best practices in family planning and reproductive health in Pakistan. Pathfinder plans to work with White Ribbon Alliance-Pakistan to address the systemic barriers that prevent more widespread use of FP/RH services by ensuring that key decision makers, front line providers and civil society are aware of

	project title	Short description	long description
			evidence-based best practices that will increase access and demand for FP/RH services and improve the integration of these services with other maternal, child, and neonatal health (MNCH) programs. to support integration of FP into postpartum, post abortion, and child health services for the urban poor by updating policy makers, public and private sector providers, professional organizations and civil society for Pakistan
22	CONGENITAL SYPHILIS EN THE DEPARTMENT HEADQUATERS OF MISIONS (ARGENTINA). INVESTIGATION ON EPIDEMIOLOGY WITH INTERVENTIONS OF PREVENTION AND PROMOTION TO WOMEN IN RISK.	MEDICAL RESEARCH	SÍFILIS CONGÉNITA EN EL DEPARTAMENTO CAPITAL DE MISIONES (ARGENTINA). ESTUDIO EPIDEMIOLÓGICO CON INTERVENCIONES DE PREVENCIÓN Y PROMOCIÓN A MUJERES EN RIESGO.
23	Karitane - Developing Evidence-based Parenting & Perinatal Services for Chinese Families	KARITANE - DEVELOPING EVIDENCE-BASED PARENTING & PERINATAL SERVICES FOR CHINESE FAMILIES	[blank]
24	Landscape Analysis of Maternal and Perinatal Infections	LANDSCAPE ANALYSIS OF MATERNAL AND PERINATAL INFECTIONS	To identify and recommend investment opportunities in diagnostic technologies aimed at improving management and control of infections linked to maternal and perinatal mortality through a comprehensive evaluation of the literature and primary research in three high-burden countries. Landscape diagnostic tools and treatments for maternal infections
25	Malaria in Pregnancy Consortium	MALARIA IN PREGNANCY CONSORTIUM	The project aims to identify the optimal combination of existing and new interventions for the treatment and prevention of malaria in pregnancy (MiP) that will enable malaria control program managers to implement improved, evidence-based interventions and strategies to control MiP in different transmission settings in Africa, Asia and Latin America. The projectÆs primary goals are to identify new safe and effective antimalarial drugs for the treatment and prevention of MiP; to determine the optimal combinations of treatment, vector control, and intermittent preventive treatment strategies; and to determine optimal ways of scaling up the use of existing and new tools to control MiP. We will establish a consortium to facilitate effective communication between stakeholders to ensure a comprehensive, standardized, and systematic approach to research, to provide advocacy, and to serve as a resource centre so that new ways of controlling MiP are found and implemented as speedily and effectively as possible. to evaluate new and existing drug-based interventions and vector control measures to improve the control of MiP across the range of malaria transmission settings in order to save the lives of mothers and their infants
26	Maternal and Neonatal Directed Assessment of Technology (MANDATE)	MATERNAL AND NEONATAL DIRECTED ASSESSMENT OF TECHNOLOGY (MANDATE)	In low-resource settings, maternal, fetal and neonatal mortality rates are unacceptably high, but there is no global agreement on how to prioritize the development of potential technological interventions to help reduce these mortalities or improve general maternal and infant health. This proposed work will inform the development of appropriate, effective technologies for improved maternal and neonatal health care by providing an evidence-based framework comparing potential health impact and other critical factors to guide the global health communityÆs development of these technologies directed toward both prevention and treatment of conditions leading to mortality. There are multiple audiences for the information that will result from the work, including funding agencies, developers of technology, as well as health providers and health policy makers. Maternal and Neonatal Technologies Evaluation Initiative-The project will create a framework and assess potential new techologies for maternal and neonatal health. RTI will provide a decision modeling tool and recommendations to the Bill & Melinda Gates Foundation (BMGF) for prioritizing investment in new, approrpiate technologies.
27	Measurement, learning and evaluation (MLE) initiative: independent impact evaluation of	MEASUREMENT, LEARNING AND EVALUATION INITIATIVE: INDEPENDENT	to learn, improve on, scale and sustain impact through harmonized, synthesized, and applied results measurement, while providing the needed evidence to influence and leverage global

	project title	Short description	long description
	foundation investments in MNCH in India, Ethiopia, and Nigeria	IMPACT EVALUATION OF FOUNDATION INVESTMENTS IN MNCH	action and optimize our critical path
28	Recherche médicale	MEDICAL RESEARCH	Santé de la mère et de l'enfant en milieu tropical : épidémiologie génétique et périnatale
29	Preventing maternal and neonatal infections: A randomized trial evaluating whether chlorhexidine disinfection of the birth canal during labor prevents maternal and neonatal sepsis in a population with high rates of maternal HIV infection, Soweto, South A	PREVENTING MATERNAL AND NEONATAL INFECTIONS.	To determine through a clinical trial whether disinfection of the birth canal during labor and newborn at birth prevents maternal and newborn bacterial infections in resource poor settings with high maternal and neonatal morbidity and mortality. to determine whether application of a safe, cheap disinfectant to the birth canal during labor and newborn at birth prevents maternal and newborn bacterial infections in resource poor settings with high maternal and neonatal morbidity and mortality
30	Éducation et formation plurisectorielles	MULTISECTOR EDUCATION/TRAINING	Atelier « Jeunes Equipes Associées à l'IRD » à Bamako-30 nov/3 déc 2010. Recherche clinique sur la santé maternelle et périnatale au Mali
31	PCI. NON-INVASIVE PRENATAL DIAGNOSIS OF HAEMOGLOBINOPATHIES AND MONITORING OF PRE-ECLAMPSIA: ISOLATION AND GENETIC CHARACTERISATION OF CIRCULATING CE	PERSONNEL DVPT: POP. & REPRO HEALTH	NON-INVASIVE PRENATAL DIAGNOSIS OF HAEMOGLOBINOPATHIES AND MONITORING OF PRE-ECLAMPSIA: ISOLATION AND GENETIC CHARACTERISATION OF CIRCULATING CELL-FREE FETAL DNA- ¿AFRONIPPED¿
32	PIC 2007 Projet de recherche en chimie hématologique et de lutte contre la drépanocytose	PIC 2007 PROJET DE RECHERCHE EN CHIMIE HÉMATOLOGIQUE ET DE LUTTE CONTRE LA DRÉPANOCYTOSE	CIUF PIC 2007 Projet de recherche en chimie hématologique et de lutte contre la drépanocytose - RDC Parmi les pathologies du globule rouge, la drépanocytose est une maladie chronique, sévère, ponctuée pour beaucoup de malades par des épisodes aigus. Dans les pays industrialisés, l'introduction du dépistage néonatal a permis d'identifier très précocement les nouveau-nés atteints, d'instaurer une prophylaxie et des vaccinations, mais aussi de donner des conseils de prise en charge aux parents. Ces traitements et conseils limitent les épisodes aigus. Ces mesures simples, peu onéreuses, peuvent être appliquées en RDC et permettre de diminuer de manière significative la morbidité et la mortalité liées à cette affection.L'objet du projet est la mise en place d'un laboratoire de référence et de recherche en chimie hématologique et l'amélioration du dépistage et de la prévention de la drépanocytose.La recherche en chimie hématologique vise l'élucidation de l'origine des maladies héréditaires du globule rouge. Le projet s'assigne donc l'objectif de rechercher les réponses à tous les cas de maladies du globule rouge rencontrées au Congo. Ce qui permettra, à terme, d'en faciliter le diagnostic et d'en améliorer les traitements.Le projet a donc deux mission essentielles :La création d'un laboratoire de référence et la recherche en chimie hématologique. Ce laboratoire de référence dans le dépistage et le diagnostic des maladies du globule, grâce à l'acquisition d'équipements analytiques performants, sera capable d'effectuer avec assurance
33	Promote evidence-based decision making in designing maternal, neonatal and child health interventions in low- and middle-income countries.	PROMOTE EVIDENCE-BASED DECISION MAKING IN DESIGNING MATERNAL, NEONATAL AND CHILD HEALTH INTERVENTIONS IN LOW- AND MIDDLE-INCOME COUNTRIES.	programs that focus on maternal and child survival. The funds will build a tool that utilizes knowledge about the impact of interventions on child and maternal mortality to identify implementation constraints of the current health system, identifies those constraints should be removed to optimize expected health outcomes, then, estimates the marginal costs of overcoming these constraints. The tool will be developed within the Spectrum software package which includes as its base a demographic engine that can be used to project population and demographic information. This tool will be used to assist in setting targets for demonstrated high impact interventions, their expected impact, cost per life saved as well as the funding requirements for any additional costs. The tool uses existing information available for selected interventions to identify the bottlenecks û that is, the weakest links in the chain of conditions and the various options to address them. Ultimately, this project refines the previous work on impact of interventions done under the auspices of the Child Health Epidemiology Reference Group and the Marginal Budgeting for Bottlenecks approach, as a basis to analyze system wide bottlenecks

	project title	Short description	long description
			to health care, their underlying causes and the operational strategies to remove them. to promote evidence-based decision making in designing maternal, neonatal and child health interventions in low- and middle-income countries
34	Providing the evidence base for improving the nutritional status of infants and lactating women in resource-poor settings using lipid-based micronutrient-rich supplements (LNS)	PROVIDING THE EVIDENCE BASE FOR IMPROVING THE NUTRITIONAL STATUS OF INFANTS AND LACTATING WOMEN.	Our main goal is to provide the scientific evidence to support the broad use of low-cost LNS to enhance the nutritional status of women and children. We will address three broad research questions: (1) How does LNS given to infants who are not breast-fed in the second six months of life affect their nutritional status, growth and health during that time period? (2) How does LNS given to lactating women affect the nutritional status, growth and health of their breastfeeding infant? (3) How does LNS given to lactating women affect their own health and nutritional status? to provide the evidence base for improving the nutritional status of lactating women and their infants in resource-poor settings with high HIV prevalence by using lipid-based micronutrient-rich supplements (LNS)
35	Lancet series on Integration of Maternal, Newborn and Child Health	REPRODUCTIVE HEALTH CARE	Financial support to Lancet Series on Integration of Maternal, Newborn and Child Health (MNCH) which will be published in the first half of 2008 , and has been conceptualized in close consultation with the Chief Editor of Lancet and several opinion leader
36	IMT - CENTRE MURAZ SANTÉ MATERNELLE ET NOUVEAU-NÉS, PALUDISME, NUTRITION	SANTÉ MATERNELLE ET NOUVEAU-NÉS, PALUDISME, NUTRITION	IMT - CENTRE MURAZ SANTÉ MATERNELLE ET NOUVEAU-NÉS, PALUDISME, NUTRITION
37	Save lives, Save Limbs! Cambodian Resource Center for Rural Health	SAVE LIVES, SAVE LIMBS! CAMBODIAN RESOURCE CENTER FOR RURAL HEALTH	Action research center that works to improve access to basic health services in rural Cambodia by: developing chains-of-survival for victims of trauma (mine victims) to reduce mortality rates to 10 percent in target areas; developing a Delivery Life Support model that reduces maternal and neonatal mortality; establishing teams of Khmer medical researchers at rural hospitals; establishing a research training center in Battambang.
38	Saving Grace - A Night of Hope	SAVING GRACE - A NIGHT OF HOPE	Table Sponsorship at the gala, which will be the organization's biggest fundraiser of 2009. The funds will support the organization's programs and services including new research studies, the redevelopment of their web site, and investing in new evidence-based patient education materials to reach women of all literacy levels. Preeclampsia, high blood pressure during pregnancy, is responsible for the death of an estimated 76,000 mothers and 500,000 babies annually, worldwide. The condition is a focus of the MNCH strategy, as new information on etiopathology, early screening, prevention, and management is forthcoming and will trigger policy and program updates. This gala will be the organization's biggest fundraiser of 2009. The funds will support the organization's programs and services including new research studies, the redevelopment of their web site, and investing in new evidence-based patient education materials to reach women of all literacy levels. Preeclampsia, high blood pressure during pregnancy, is responsible for the death of an estimated 76,000 mothers and 500,000 babies annually, worldwide. The condition is a focus of the MNCH strategy, as new information on etiopathology, early screening, prevention, and management is forthcoming and will trigger policy and program updates.
39	SAVING MATERNAL AND NEWBORN LIVES - ZVITAMBO - THE RESEARCH INSTITUTE OF THE MCGILL UNIVERSITY HEALTH CENTRE	SAVING MATERNAL AND NEWBORN LIVES - ZVITAMBO - THE RESEARCH INSTITUTE OF THE MCGILL UNIVERSITY HEALTH CENTRE	To maintain access to family planning services and to protect the lives of Mothers and Newborns affected by HIV and Aids
40	Simplified Regimens for Management of Possible Serious Bacterial Infections in Neonates and Young Infants for Use in Outpatient and Community Settings: A multi-	SIMPLIFIED REGIMENS FOR MANAGEMENT OF POSSIBLE SERIOUS BACTERIAL INFECTIONS IN NEONATES AND YOUNG INFANTS.	This project aims to evaluate the safety and efficacy of simplified antibiotic treatment regimens for young infants with possible serious bacterial infections and to use the evidence to inform global policy. Such policy would result in increased coverage with treatment and improved neonatal and infant survival in low- and middle-income countries. Evaluate the impact of topical

	project title	Short description	long description
	centre Randomized Controlled Trial in Africa		application of emollient(s) for prevention of neonatal infections and mortality
41	A Live Recombinant Attenuated Salmonella Anti-Pneumococcal Vaccine for Newborns	A LIVE RECOMBINANT ATTENUATED SALMONELLA ANTI-PNEUMOCOCCAL VACCINE FOR NEWBORNS	The overall goal of our project is to design, construct and evaluate an oral recombinant attenuated Salmonella vaccine to induce protective immunity to infants and young children against diverse strains of Streptococcus pneumoniae encountered in the developing world. Genetically modified strains of S. typhi will be used for human trials and genetically modified strains of S. typhimurium will be used to fully develop all the strategies and validate vaccine safety and efficacy in mice. to develop a live, recombinant, attenuated Salmonella anti-pneumococcal vaccine for newborns
42	Strengthening the evidence base for the epidemiology of maternal, neonatal and child conditions	STRENGTHENING THE EVIDENCE BASE FOR THE EPIDEMIOLOGY OF MATERNAL, NEONATAL AND CHILD CONDITIONS	to support priority areas for continued work by the Child Health Reference Epidemiology Group (CHERG) on data access, methodology, and estimating morbidity and mortality for neonatal, maternal and child conditions
43	Sure Start (Community Health Solutions in India)	SURE START (COMMUNITY HEALTH SOLUTIONS IN INDIA)	The overall purpose of Safe Passages is to develop, demonstrate and refine a model of channeling financial and technical resources to the community level, in order to support effective community action on public health priorities. The specific public health goal to be addressed is the reduction of newborn and maternal morbidity and mortality in India. to demonstrate a demand creation model to enhance NRHM services that saves newborn lives and reduces maternal mortality and morbidity in communities in India (Uttar Pradesh and Maharashtra)
44	TLR8 agonists: adjuvants to enhance neonatal vaccination.	TLR8 AGONISTS: ADJUVANTS TO ENHANCE NEONATAL VACCINATION.	Newborns and infants are highly susceptible to lmidazoquinolinesacvateTLR8 infection resulting in over I million deaths per year worldwide. As birth is the most reliable point of healthcare contact, neonatal vaccines are a global health priority. However, newborns have immature immunity that limits their responses to most stimuli, frustrating efforts to protect this vulnerable population. There is therefore an unmet medical need to develop more N N effective neonatal vaccines. Our idea is that novel immuno-stimulatory molecules (;imidazoquinolines ;, Fig. I) that effectively activate white blood cells of newborns may serve as effective neonatal vaccine adjuvants, allowing effective vaccination at birth. to enhance neonatal vaccination
45	to demonstrate and leverage uptake of scalable models of delivery and immediate newborn care in rural Ethiopia	TO DEMONSTRATE AND LEVERAGE UPTAKE OF SCALABLE MODELS OF DELIVERY AND IMMEDIATE NEWBORN CARE IN RURAL ETHIOPIA	To demonstrate a community-oriented model to improve maternal and newborn health care in rural Ethiopia and position it for scale up. Working collaboratively with the Ministry of Health (MOH) and Regional Health Bureaus (RHB), this learning project will use a ælead woreda approach' in an effort to improve this situation in two regions of the country and to demonstrate its broader scalability. In keeping with the GatesÆ model of change (more, better, more equitable), this community-oriented approach will strengthen provider capacity at local and regional levels, increase local demand from women and families for enhanced MNH care, and, using locally designed and tested solutions, and overcome key challenges to in-home provision of a defined package of evidence-based MNH care practices during the critical birth-to-48 hours window. The projectÆs organization, operation, and programs are geared to promote broader uptake of the model, to strengthen federal and local capacity to do so, and to provide a learning-based toolkit to facilitate scale-up. to demonstrate and leverage uptake of scalable models of delivery and immediate newborn care in rural Ethiopia
46	to further develop and accelerate antimalarial discovery and development projects	TO FURTHER DEVELOP AND ACCELERATE ANTIMALARIAL DISCOVERY AND DEVELOPMENT PROJECTS	The present portfolio is largely directed at the priority goal of developing drugs for uncomplicated P.falciparum malaria with one clinical project focused on providing a new treatment for complicated severe malaria and another for P.vivax malaria. As the portfolio matures, and products are launched, the work is expanding to optimize these medicines for use in treatments leading to the eradication of the disease. Particularly, studies on use of ACTs for the intermittent preventive treatment for pregnant women (IPTp) or in infants (IPTi) are planned. These require additional safety data and efficacy data as therapeutics before the key work on prevention can start. The early stage portfolio is being focused on the needs beyond 2010, in a malaria

	project title	Short description	long description
			community where a choice of ICH GCP quality ACTs are available. Specifically we are looking at innovative drugs for the highly desirable æone dose curesÆ, gametocidal and transmission blocking drugs which are a key for elimination, and new approaches to minimize the impact of potential resistance to Artemesinins. MMV and their partners have four new combination drugs with the potential to launch over the next two years. Each has a distinct footprint in the treatment of malaria. The aim is that the public sector target price for a full course of treatment with these innovative new combination therapies is 1 US dollar for adults and 50c. for children, or less. We also hope to achieve the lowest possible private sector prices. to further develop and accelerate antimalarial discovery and development projects. Product Development Partnership
47	Use of Mobile Phones for Improvement of MNCH Care	USE OF MOBILE PHONES FOR IMPROVEMENT OF MNCH CARE	to field test in central Mozambique two mobile phone modules that prompt community health workers caring for pregnant women and newborns to assess, to take action, and to refer care in cases of complications and emergencies
48	[blank]	MEDICAL RESEARCH	To continue epidemiologic studies of reproductive and developmental outcomes in Denmark as related to fetal alcohol syndrome

Table 15. Titles and descriptions of research activities exclusively benefitting newborns.
This is an exhaustive list of the record descriptions categorised as research projects exclusively benefitting newborns. The 20 sets of descriptions here reflect 26 different records, as some records include identical descriptions.

	project title	Short description	long description
1	Aetiology of Neonatal Infection in South Asia (ANISA)	AETIOLOGY OF NEONATAL INFECTION IN SOUTH ASIA (ANISA)	for etiology (bacterial and viral agents) of neonatal sepsis in Asia and Africa
2	Analysis of landscape and potential impact of diagnostics for neonatal infections	ANALYSIS OF LANDSCAPE AND POTENTIAL IMPACT OF DIAGNOSTICS FOR NEONATAL INFECTIONS	to review the existing and emerging biomarkers and diagnostic tests for neonatal infections/sepsis in the context of developing countries, and to model the potential health impact that diagnostic tests could achieve by informing appropriate treatment deci
3	CHILD HEALTH KNOWL'PROG	CHILD HEALTH KNOWL'PROG	The Impact of a community-based participatory intervention to improve essential newborn care in rural Nepal: a randomised controlled trial
4	Creation of a highly stable pulmonary surfactant replacement	CREATION OF A HIGHLY STABLE PULMONARY SURFACTANT REPLACEMENT	to develop a dry powder pulmonary surfactant formulation for the treatment of respiratory distress syndrome in newborns
5	Efficacy of newborn vitamin A supplementation in improving child survival	EFFICACY OF NEWBORN VITAMIN A SUPPLEMENTATION IN IMPROVING CHILD SURVIVAL	Project Goal: To inform global policy on the use of neonatal vitamin A supplementation for reducing infant mortality in low- and middle-income countries. Objectives: 1) To conduct randomized controlled trials, two in Africa and one in south Asia, to determine the: - efficacy of neonatal vitamin A supplementation given in the first 48 hours of life in reducing mortality in the first half of infancy - efficacy of the above intervention in reducing severe morbidity in the first half of infancy - safety of the above intervention 2) To conduct studies to better understand the potential biological mechanisms through which neonatal vitamin A supplementation can have an impact on infant survival. To inform global policy on the use of neonatal vitamin A supplementation for reducing infant mortality, by conducting three randomized controlled trials and better understanding the biological mechanisms of action
6	Enabling Vaccines in Newborns Through Improved Understanding of Immune-Triggering Events in Neonatal Cord Blood Cells	ENABLING VACCINES IN NEWBORNS THROUGH IMPROVED UNDERSTANDING OF IMMUNE-TRIGGERING EVENTS IN NEONATAL CORD BLOOD CELLS	to test novel technologies for developing more effective neonatal vaccines, which can be applied across a broad range of vaccinal antigens,to improve infant health and survival, particularly in low income countries and the world's poorest communities, whe
7	R7426:ESSENTIAL NEWBORN CARE	ESSENTIAL NEWBORN CARE	a randomised controlled trial to assess the impact of a community-based participatory intervention to improve essential newborn care (ENC) in Rural Nepal: a randomised controlled trial.
8	Etiology (bacterial and viral agents) of neonatal sepsis in Asia and Africa	ETIOLOGY (BACTERIAL AND VIRAL AGENTS) OF NEONATAL SEPSIS IN ASIA AND AFRICA	This study will substantially help to improve newborn survival world-wide by providing data on etiology of community acquired newborn infections and antibiotic sensitivity patterns of the bacterial isolates which will, in turn, guide design of strategies to more effectively prevent and treat newborn infections. In addition, during the study period, the project will ensure treatment of infants identified with infection which is expected to save lives at the study sites. Further, the study will train a large number of local community health workers, mostly women, who will remain in the respective

	project title	Short description	long description
9			communities and continue to contribute to improving the care seeking behavior of the people in the study population. The study will create awareness in the study area and empower women with knowledge to save newborn lives. Etiology (bacterial and viral agents) of neonatal sepsis in Asia and Africa
9	Evaluate the impact of topical application of emollient(s) for prevention of neonatal infections and mortality (India)	EVALUATE THE IMPACT OF TOPICAL APPLICATION OF EMOLLIENT(S) FOR PREVENTION OF NEONATAL INFECTIONS AND MORTALITY (INDIA)	To develop and evaluate the impact of a potentially scalable community-based intervention of emollient therapy and/or improved skin care practices on neonatal mortality in a high mortality resource poor setting in India. Evaluate the impact of topical application of emollient(s) for prevention of neonatal infections and mortality (India)
10	Impact of chlorhexidine cord cleansing for prevention of neonatal mortality in Pemba, Tanzania	IMPACT OF CHLORHEXIDINE CORD CLEANSING FOR PREVENTION OF NEONATAL MORTALITY IN PEMBA, TANZANIA	To evaluate the efficacy of cord cleansing with chlorhexidine in the first week of life for reducing neonatal mortality and morbidity. The study would provide a proof of principle for an intervention that could easily be scaled up and incorporated into preventive health care in sub-Saharan Africa, impacting a part of the 1.2 million neonatal deaths and 27 million years of life lost every year in sub-Saharan Africa. Chlorhexidine being inexpensive, with a strong safety record, is an ideal intervention for low-resource communities. Chlorhexidine is currently included in the WHO Essential Drug List. It is recommended as preferred agent if an antiseptic is to be used on the cord. to evaluate the efficacy of chlorhexidine to clean umbilical cord of neonates in first 10 days for reduction in neonatal mortality and Omphalitis: a community-based randomized, double-masked controlled trial in Pemba, Tanzania
11	Impact of chlorhexidine cord cleansing for prevention of neonatal mortality in Zambia	IMPACT OF CHLORHEXIDINE CORD CLEANSING FOR PREVENTION OF NEONATAL MORTALITY IN ZAMBIA	The aim of this project is to conduct a randomized, controlled study designed to reduce neonatal mortality in a sub-Saharan African country in order to provide evidence for decision making in designing neonatal health interventions in low- and middle-income countries. to evaluate the effectiveness of 4% chlorhexidine umbilical cord wash compared to dry cord care for the reduction of neonatal mortality in southern province, Zambia
12	Lactoferrin for prevention of sepsis in young infants	LACTOFERRIN FOR PREVENTION OF SEPSIS IN YOUNG INFANTS	To test whether providing newborns with daily oral supplements of a key milk protein, Lactoferrin,can protect them against sepsis during the critical early days in life.
13	Linking Innate and Specific Immunity to Develop Single Dose Vaccines for Neonates	LINKING INNATE AND SPECIFIC IMMUNITY TO DEVELOP SINGLE DOSE VACCINES FOR NEONATES	Currently, very young children (neonates) are the most vulnerable of all members of society to infectious diseases. This vulnerability is partially due to the way neonates respond or donÆt respond to current vaccines. We feel that by developing novel formulations for currently available vaccines, we can improve the quality of the immunological responses of neonates so that they will be protected from infection. Our approach will be to use novel compounds to stimulate immunity, but, more importantly, formulate these compounds with vaccines in such a way as to stimulate protection at the site of entry of the pathogen (mucosal surfaces), as well as systematically. Secondly, by delivery, the vaccines to mucosal surfaces, we hope to replace needles as a method of delivery. These factors combined should dramatically improve vaccine coverage (compliance) and, thereby, have a significant impact on both the morbidity and mortality of children around the world. to develop single dose vaccines for neonates by linking innate and specific immunity

	project title	Short description	long description
14	Newborn cry-based diagnosis system	NEWBORN CRY-BASED DIAGNOSIS SYSTEM	to design and test a diagnostic tool using computer acoustical analysis of newborn cries to complement conventional diagnostic techniques in detecting medical conditions such as asphyxia, hypoglycemia, and infections
15	Rapid development of a simplified antibiotic regimen for newborn infection management in first-level facility and community settings.	RAPID DEVELOPMENT OF A SIMPLIFIED ANTIBIOTIC REGIMEN FOR NEWBORN INFECTION MANAGEMENT IN FIRST-LEVEL FACILITY AND COMMUNITY SETTINGS.	to support rapid development of a simplified antibiotic regimen for newborn infection management in first-level facility and community settings
16	Targeting mTOR signaling to prevent preterm birth	TARGETING MTOR SIGNALING TO PREVENT PRETERM BIRTH	to test the hypothesis that the protein mTor, which regulates cell growth and survival, plays a critical role in premature uterine aging that lead to preterm birth, difficult labor and fetal death
17	Analysis of landscape and potential impact of diagnostics for neonatal infections	ANALYSIS OF LANDSCAPE AND POTENTIAL IMPACT OF DIAGNOSTICS FOR NEONATAL INFECTIONS	To review the existing and emerging biomarkers and diagnostic tests for neonatal infections/sepsis in the context of developing countries, and to model the potential health impact that diagnostic tests could achieve by informing appropriate treatment decisions to review the existing and emerging biomarkers and diagnostic tests for neonatal infections/sepsis in the context of developing countries, and to model the potential health impact that diagnostic tests could achieve by informing appropriate treatment decisions
18	The International Fetal and Newborn Growth Consortium for the 21st Century (INTERGROWTH-21st)	THE INTERNATIONAL FETAL AND NEWBORN GROWTH CONSORTIUM FOR THE 21ST CENTURY (INTERGROWTH-21ST)	The project aims to develop scientifically robust clinical tools to assess fetal growth and the nutritional status of newborn infants, as adjuncts to the recently produced WHO charts for children aged 0 to 5. These will be incorporated into national and international maternal and neonatal programs, and they will be used to monitor and evaluate maternal wellbeing, infant health and nutrition at a population level. To achieve these objectives, primary data will be collected in 10 countries on a population based sample of healthy pregnant women. The tools will describe how fetuses and newborns should grow in all countries rather than the more limited objective of past growth references which describe how they have grown at specific times and locations. They will allow for evidence based evaluation of nutritional status at birth and measurement of the impact of preventive and treatment interventions in the community. to develop international Fetal and Newborn Growth Standards and to relate these to neonatal health risk
19	The Lancet Stillbirth Series	THE LANCET STILLBIRTH SERIES	to produce a series of papers for The Lancet placing stillbirths as a priority within the context of maternal and child survival, bringing together epidemiology, evidence for interventions, costing estimates, and policy analysis to promote action and guid
20	Tools, Technologies and Approaches to Improve Newborn Health and Survival	TOOLS, TECHNOLOGIES AND APPROACHES TO IMPROVE NEWBORN HEALTH AND SURVIVAL	Saving Newborn Lives will advance global capacity to reduce neonatal mortality by refining evidence-based, low cost interventions and demonstrating how to integrate and scale up these interventions within existing health systems in selected countries in sub-Saharan Africa and South Asia. We will focus on the three major killers of newbornsùinfections, birth asphyxia, and prematurity/low birth weightù that occur in the first week of life and strategies for reaching the communities where most newborns are born and die. to test and evaluate a critical set of newborn health care

	project title	Short description	long description
			tools & technologies

Table 16. Inter-rater reliability.
The following tables present details of the reliability of coding. The term “value” refers to the absolute monetary value of projects; negative grants, which reflect net returns of monies to the donor which might happen following a project which spends below its allocated budget, and therefore included in this analysis as positive values.

a. Inter-rater reliability

1. Records exclusively benefitting newborns

	N	%	Value	%
Agree	4509	**98.4%**	2,545.6	**98.1%**
Disagree	75	1.6%	50.3	1.9%
Total	4584	**100.0%**	2,596.0	**100.0%**
Kappa*	0.91		0.81	

*Weighted and unweighted kappa values round to the same figure at two decimal places

2. Records supporting research

	N	%	Value	%
Agree	4461	**97.3%**	2,424.0	**93.4%**
Disagree	123	2.7%	172.0	6.6%
Total	4584	**100.0%**	2,596.0	**100.0%**
Kappa*	0.47		0.59	

*Based on analysis of 2 x 2 matrix of Research vs. Not research as misclassification was assessed based on whether includes or exclusively benefits newborns.

b. Details of raters' coding

1. Records exclusively benefitting newborns by number

	**Coder 2**		
**Coder 1**	Exclusively newborns	Includes newborns	Misclassified
Exclusively newborns	**441**	18	2
Includes newborns	10	**4063**	5
Misclassified	0	40	**5**

2. Records supporting research by number

	**Coder 2**		
**Coder 1**	Research	Not Research	Misclassified
Research	**35**	61	
Not Research	15	**4427**	
Misclassified			**46**

3. Records exclusively benefitting newborns by absolute value

	**Coder 2**		
**Coder 1**	Exclusively newborns	Includes newborns	Misclassified
Exclusively newborns	**109.7**	6.7	0.7
Includes newborns	1.9	**2,433.8**	0.6
Misclassified	0.0	40.4	**2.1**

4. Records supporting research by absolute value

	**Coder 2**		
**Coder 1**	Research	Not Research	Misclassified
Research	**102.3**	127.8	
Not Research	2.5	**2,320.6**	
Misclassified			**42.8**

c. Final agreed coding

1. Records exclusively benefitting newborns

	N	%	Value	%
Exclusively newborns	456	9.9%	113.6	4.4%
Includes newborns	4082	89.0%	2,437.8	94.0%
Misclassified	46	1.0%	42.8	1.7%
Total	4584	100.0%	2,594.2	100.0%

2. Records supporting research

	N	%	Value	%
Research	88	1.9%	175.2	6.8%
Not Research	4450	97.1%	2,376.2	91.6%
Misclassified	46	1.0%	42.8	1.7%
Total	4584	100.0%	2,594.2	100.0%

Part III: References

1. UN Population Division (2009) World Population Prospects: The 2008 Revision Population Database. 11 March 2009 ed: Department of Economic and Social Affairs.
2. OECD (2010) Information Note on the DAC Deflators. Paris: Organisation for Economic Co-operation and Development.
3. Pitt C, Greco G, Powell-Jackson T, Mills A (2010) Countdown to 2015: assessment of official development assistance to maternal, newborn, and child health, 2003-08. Lancet 376: 1485-1496.
4. OECD (2007) Reporting Directives for the Creditor Reporting System. Paris: Organisation for Economic Co-operation and Development
5. Evaluation IfHMa (2010) Financing global health 2010: Development assistance and country spending in economic uncertainty: Methods Annex. Seattle, WA: IHME.
6. Murray CJ, Anderson B, Burstein R, Leach-Kemon K, Schneider M, et al. (2011) Development assistance for health: trends and prospects. Lancet.
7. Institute for Health Metrics and Evaluation (2011) Financing global health 2011: Continued Growth as MDG Deadline Approaches: Methods Annex. Seattle, WA: IHME.
